# Genomic profiling reveals heterogeneous populations of ductal carcinoma in situ of the breast

**DOI:** 10.1038/s42003-021-01959-9

**Published:** 2021-04-01

**Authors:** Satoi Nagasawa, Yuta Kuze, Ichiro Maeda, Yasuyuki Kojima, Ai Motoyoshi, Tatsuya Onishi, Tsuguo Iwatani, Takamichi Yokoe, Junki Koike, Motohiro Chosokabe, Manabu Kubota, Hibiki Seino, Ayako Suzuki, Masahide Seki, Katsuya Tsuchihara, Eisuke Inoue, Koichiro Tsugawa, Tomohiko Ohta, Yutaka Suzuki

**Affiliations:** 1 Department of Computational Biology and Medical Sciences, Graduate School of Frontier Sciences, The University of Tokyo, Kashiwa-shi, Chiba Japan; 2 Division of Breast and Endocrine Surgery, Department of Surgery, St. Marianna University School of Medicine, Miyamae-ku, Kawasaki Japan; 3 Department of Breast Surgery, National Cancer Center Hospital East, Kashiwa, Chiba Japan; 4 Department of Diagnostic Pathology, Kitasato University Kitasato Institute Hospital, Minato-ku, Tokyo Japan; 5 Department of Pathology, Kitasato University School of Medicine, Minami-ku, Sagamihara Japan; 6 Department of Pathology, St. Marianna University School of Medicine, Miyamae-ku, Kawasaki Japan; 7 Division of Translational Informatics, Exploratory Oncology Research and Clinical Trial Center, National Cancer Center, Kashiwa, Chiba Japan; 8 Showa University Research Administration Center, Showa University, Shinagawa-ku, Tokyo Japan; 9 Department of Translational Oncology, St. Marianna University Graduate School of Medicine, Miyamae-ku, Kawasaki Japan

**Keywords:** Tumour heterogeneity, Prognostic markers, Surgical oncology, Breast cancer

## Abstract

In a substantial number of patients, ductal carcinoma in situ (DCIS) of the breast will never progress to invasive ductal carcinoma, and these patients are often overtreated under the current clinical criteria. Although various candidate markers are available, relevant markers for delineating risk categories have not yet been established. In this study, we analyzed the clinical characteristics of 431 patients with DCIS and performed whole-exome sequencing analysis in a 21-patient discovery cohort and targeted deep sequencing analysis in a 72-patient validation cohort. We determined that age <45 years, *HER2* amplification, and *GATA3* mutation are possible indicators of relapse. *PIK3CA* mutation negativity and PgR negativity were also suggested to be risk factors. Spatial transcriptome analysis further revealed that *GATA3* dysfunction upregulates epithelial-to-mesenchymal transition and angiogenesis, followed by PgR downregulation. These results reveal the existence of heterogeneous cell populations in DCIS and provide predictive markers for classifying DCIS and optimizing treatment.

## Abstract (web summary)

Satoi Nagasawa and Yuta Kuze et al. report a multi-omic analysis of ductal carcinoma in situ (DCIS) of the breast, including whole-exome, single-cell, and spatial transcriptome sequencing. They find that for patients under 45 years of age, *HER2* amplification and *GATA3* mutation are associated with higher risk of relapse, suggesting they could be used as predictive markers when deciding on a treatment course.

## Introduction

Noninvasive ductal carcinoma of the breast, i.e., ductal carcinoma in situ (DCIS), is an early stage of breast cancer that may potentially develop into invasive ductal carcinoma (IDC)^1,2^. Because the breast duct is anatomically free of blood or lymphatic vessels, the lesions localized in situ theoretically do not undergo metastasis. The incidence of DCIS has been increasing due to improvements in diagnostic modalities, permitting preventive surgery based on the assumption that surgical excision reduces the risk of eventual IDC development^3–5^. However, a substantial number of DCIS lesions diagnosed using the current criteria may not progress to IDC in the absence of treatment^6–11^. Clinically, DCIS can be divided into three categories: (1) unlikely to progress to IDC even without surgical treatment (low-risk or false DCIS); (2) truly precancerous IDC lesions (true DCIS); and (3) high potential for relapse as IDC even with standard treatment (high-risk DCIS). It is critical to discriminate low-risk DCIS from true DCIS to avoid unnecessary surgery, thereby improving patients’ quality of life and reducing medical costs. In contrast, comprehensive treatment should be provided for patients with high-risk DCIS to improve their prognosis. Therefore, accurate classification is crucial for directing appropriate treatment. Indeed, global clinical trials are underway in several countries to determine whether nonresection treatment is feasible for low-risk DCIS^12–14^. However, the current three-category classification system is not straightforward. The current classification depends on clinicopathological factors such as age, tumor size, presence of comedo necrosis, nuclear grade, and hormone receptor and human epidermal growth factor receptor 2 (HER2) status^15–19^, and in the absence of a global standard^20^, the evaluations vary among pathologists or institutions. A critical issue that has not been resolved is the lack of biological verification of the risk factors that contribute to the malignant transformation of DCIS in vivo. The small number of tumor specimens available for analysis and the heterogeneity of tumors have made such studies of DCIS difficult. To address this issue, we identified objective clinicopathological and genomic risk factors for DCIS relapse based on an analysis of the clinical features of 431 patients with DCIS (cohort 1) who lack any clinical trait of IDC, followed by whole-exome sequencing analysis in a discovery cohort of 21 patients with DCIS (cohort 2) and targeted deep sequencing analysis in a validation cohort of 72 patients with DCIS (cohort 3). Then, we examined the contributions of these factors to the progression of invasive cancer in vivo using spatial transcriptome sequencing (STseq) and single-cell DNA sequencing (scDNA-seq). STseq is a novel technique that provides gene expression information from pathological sections^21,22^. STseq allows gene expression profiling without losing the positional information of the different cell types that constitute tumors in vivo. For the first time, to the best of our knowledge, the combination of these sequencing techniques allowed us to determine the existence of heterogeneous cell populations in DCIS and their in vivo biological consequences.

## Results

### Determination of clinicopathological risk factors

The records of 431 patients with DCIS who underwent surgery at St. Marianna University School of Medicine from 2007 to 2012 (cohort 1) was reviewed to determine the clinical criteria for stratifying patients with low- or high-risk DCIS. The median age of patients at diagnosis was 48 years (range, 24–90 years). The median follow-up period was 6.1 years (range, 0.5–10.9 years). Twenty DCIS patients (4.6%) progressed to IDC during the follow-up period. In total, 375 patients (87%) were positive for estrogen receptor (ER) expression, and 81 (18.8%) were positive for *HER2* amplification. Univariate analysis using a Cox proportional hazards regression model was performed to assess the relationship between predictive factors and relapse-free survival (Table 1, Supplementary Data 1). Age (≥45 years vs. <45 years, determined via receiver operating curve [ROC] curve analysis, area under the curve [AUC] = 0.67, Supplementary Fig. S1) and *HER2* amplification status appeared to be significantly and independently associated with relapse in multivariate analysis, with hazard ratios (HRs) of 3.57 (95% confidence interval [CI] = 1.46–8.73, *P* = 0.0054) and 3.14 (95% CI = 1.28–7.7, *P* = 0.0123), respectively. Based on these results, we decided to use age and *HER2* amplification status as the major clinical criteria for distinguishing between clinically low- and high-risk patients in subsequent analyses. The presence/absence of comedo necrosis and nuclear atypia, which are conventional risk evaluation criteria, was excluded and used only for reference purposes.

**Table 1:** Clinicopathological factors associated with prognosis according to Cox proportional hazard model analysis.

	Univariate analysis			
Variable	No. of patients	HR	95% CI	*P*
Age at diagnosis				
≥45 years	294		1.0 (reference)	
<45 years	137	3.57	1.46–8.75	0.0053
Management				
BCS	260		1.0 (reference)	
Mastectomy	80	0.89	0.25–3.17	0.863
NSM	91	1.22	0.43–3.46	0.712
ER status				
Negative	56		1.0 (reference)	
Positive	375	0.82	0.24–2.79	0.747
PgR status				
Negative	81		1.0 (reference)	
Positive	350	0.85	0.29–2.56	0.779
HER2 amplification				
No	350		1.0 (reference)	
Yes	81	3.15	1.29–7.72	0.012
Subtype				
Luminal A like	321		1.0 (reference)	
Luminal B like	52	3.70	1.37–10.02	0.0101
pureHER2 like	33	1.91	0.42–8.63	0.3990
Triple negative	25	1.21	0.16–9.39	0.8540
Nuclear atypia				
Low grade	142		1.0 (reference)	
Intermediate grade	239	2.15	0.71–6.52	0.178
High grade	50	1.76	0.32–9.65	0.513
Comedo necrosis				
No	223		1.0 (reference)	
Yes	208	1.97	0.78–4.93	0.149
Tumor size (cm)	431	0.91	0.74–1.11	0.355

*HR* hazard ratio, *CI* confidence interval, *BCS* breast-conserving surgery, *NSM* nipple-sparing mastectomy, *ER* estrogen receptor, *PgR* progesterone receptor, *HER2* human epidermal growth factor receptor 2, *luminal A like* ER−/PR+/HER2−, *luminal B like* ER+/PR+/HER2+ or ER+/PR+/HER2−/MIB1 index high, *pure HER2 like* ER−/PR−/HER2 +, *triple negative* ER−/PR−/HER2−.

### Selection of genomic risk factors in whole-exome sequencing

**Fig. 1:**
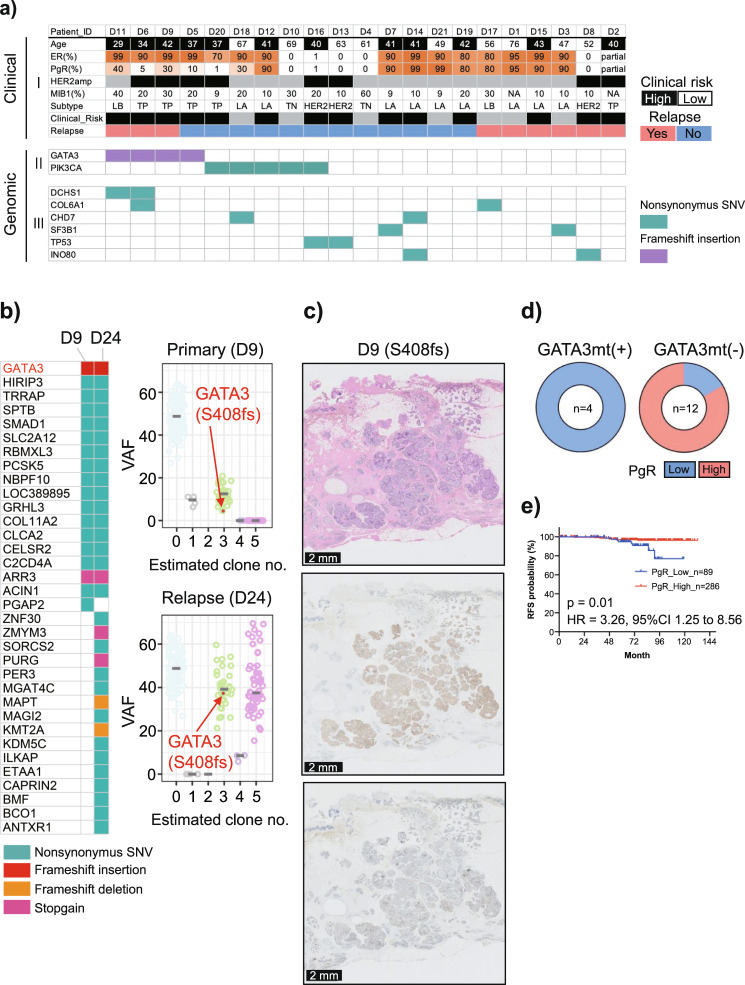
Selection of clinicopathological and genomic risk factors for relapse. **a** Results of whole-exome sequencing for 21 patients with primary pure ductal carcinoma in situ (DCIS). I: The clinical information included age; the percentages of estrogen receptor (ER)−, progesterone receptor (PgR)−, and MIB1-positive cells determined by immunohistochemistry (IHC); human epidermal growth factor receptor 2 (*HER2*) expression status; and relapse status. Patients were subdivided into molecular phenotypes using IHC surrogates (luminal A [ER−/PR+/HER2−], luminal B [ER+/PR+/HER2+ or ER+/PR+/HER2−/MIB1 index high], HER2 [ER−/PR−/HER2+], and TN [ER−/PR−/HER2−]). Clinical risk estimated by age and *HER2* expression status based on the results in Table 1 is shown. II: *GATA3* and *PIK3CA* mutations are shown (red and blue, respectively). Only the results for nonsynonymous mutations are shown. In total, 4 of 21 (19%) patients carried nonsynonymous *GATA3* mutations. Nonsynonymous *PIK3CA* mutations were detected in 5 of 21 (24%) patients. III: The genes that overlapped among patients are shown. Only the results for nonsynonymous mutations are shown. **b** Comparison of *GATA3* mutations between primary and relapse tumors. Left: Comparison of mutations determined via whole-exome sequencing of the primary (D9) and matched relapse lesions (D24) are shown. The same *GATA3* mutation (S408fs) was detected in both lesions. Right: Results of subclone analysis obtained using PyClone software are shown. The findings suggest that the subclone carrying the *GATA3* mutation also existed in the relapse lesion. VAF variant allele frequency. **c** Shows HE (upper panel), ER (middle panel), and PgR (lower panel) staining for patient D11 with a *GATA3* frameshift mutation (S408fs). Although ER expression was positive, PgR expression was decreased. Scale bars: 2 mm. HE hematoxylin and eosin, VAF variant allele frequency. **d** Pie charts depicting PgR expression assessed via IHC in 16 ER-positive patients with and without *GATA3* mutations. IHC revealed that in patients with ER-positive DCIS, *GATA3* mutation was significantly associated with reduced PgR expression (*P* = 0.0192 by Fisher’s exact test). **e** Kaplan–Meier curve for 375 patients with ER-positive DCIS according to PgR expression. High PgR expression was indicated by positive immunostaining in ≥70% of cells, and low expression was indicated by positive staining in ≤60% of cells. The prognosis of ER-positive patients with low PgR expression was significantly worse than that of patients with high PgR expression (*P* = 0.01 by log-rank test, hazard ratio = 3.26, 95% confidence interval = 1.25–8.56).

To improve the accuracy of the criteria for distinguishing between low- and high-risk DCIS based on clinical evaluation, we attempted to identify genomic factors. As a discovery cohort (cohort 2), we evaluated 21 patients with DCIS who had been randomly selected with a matched intrinsic subtype and relatively high relapse rate. The clinicopathological features of patients in cohort 2 are shown in Supplementary Table S1. Whole-exome libraries were constructed from tissue samples obtained via microdissection (Supplementary Fig. S2) and subjected to sequence analysis at a sufficient sequencing depth (averaging ×207, Supplementary Data 2). Matched normal breast tissues were sequenced (average depth, ×109.1) to distinguish germline variants from somatic mutations. The mutation graph obtained is shown in Fig. 1a.

*GATA3* and *PIK3CA* mutations were the most commonly detected mutations in the cohort and were present in four (19%) and five patients (24%), respectively. Of the four patients with *GATA3* mutations, three experienced IDC relapse. The odds ratio (OR) for relapse for *GATA3* mutation positivity versus *GATA3* mutation negativity was 5.5 (95% CI = 0.63–76.7). To further investigate the significance of *GATA3* mutation in relapse, samples from patients with relapsed tumors were additionally analyzed to determine the mutation status in comparison with their paired primary DCIS. Importantly, two of the four patients harbored the same *GATA3* mutation in their primary and relapsed tumors (Fig. 1b, Supplementary Fig. S3), suggesting that relapse was associated with a *GATA3*-mutated clone from the primary lesions. This finding was supported by the results of computational modeling performed to detect cancer evolution using the PyClone program^23^. Although the HR was not significant, based on these results, we considered *GATA3* mutation to be a candidate genomic high-risk factor in the discovery cohort.

It was recently reported that *GATA3* variants with altered DNA-binding capacity led to reduced expression of a gene set containing the progesterone receptor (*PgR*) gene and increased expression of genes involved in epithelial-to-mesenchymal transition (EMT), such as cell movement and cell invasion pathways^24^. We, therefore, examined PgR protein expression via immunohistochemical analysis in 16 patients who were ER-positive, including the four patients who harbored *GATA3* mutations. PgR expression was downregulated in all four patients with *GATA3*-mutated DCIS but downregulated in only two of the remaining 12 patients (Fig. 1c, d, Supplementary Fig. S4). We determined that *GATA3* mutation was associated with reduced PgR expression (Supplementary Table S2, *P* = 0.0192 by Fisher’s exact test). To investigate the association between prognosis and PgR expression in patients with ER-positive DCIS, we examined 375 patients with ER-positive DCIS among the clinical cohort of 431 patients. PgR expression was determined using ROC curve analysis, with an AUC of 0.66 (Supplementary Fig. S5). ER-positive patients with low PgR expression (≤60%) had a significantly higher incidence of relapse than those with high PgR expression (Fig. 1e; *P* = 0.01, HR = 3.26, 95% CI = 1.25–8.56). Therefore, low PgR expression, which is routinely measured in clinical practice, likely represents a high-risk factor that may be used as a potential surrogate marker for *GATA3* mutation in patients with ER-positive DCIS.

Contrary to the findings for *GATA3* mutation, no patient with *PIK3CA* mutation experienced relapse, whereas 9 of 16 patients without *PIK3CA* mutation (56.2%) experienced relapses. Although *PIK3CA* mutations are known cancer drivers in many cancer types^25^, interestingly, they have been associated with better patient prognosis in invasive breast cancer than wild-type *PIK3CA*^26^. Thus, we identified *PIK3CA* mutation as a candidate genomic low-risk factor for DCIS in the discovery cohort.

### Validation analysis of risk factors via targeted deep sequencing

To validate the findings revealed using the clinical data set and discovery cohort, we conducted an analysis using a larger and independent validation cohort of 72 patients (cohort 3). The clinicopathological characteristics of these patients are shown in Supplementary Table. S1. To expedite sequencing, a custom sequencing panel was designed on the basis of the observed mutation spectrum of the discovery cohort, which included 180 genes (Supplementary Table S3). The median follow-up period was 5.5 years (range, 0.5–9.6 years). Of 72 patients, nine experienced relapses. In this cohort, high clinical risk, as determined using the previously identified criteria (Table 1), tended to be correlated with poor outcome, albeit without statistical significance (OR = 1.2, 95% CI = 0.31–4.5).

**Fig. 2:**
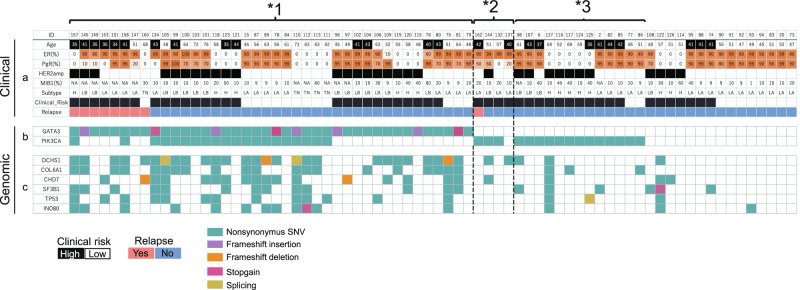
Validation of clinicopathological and genomic risk factors via targeted deep sequencing. **a** The clinical information included age; percentages of estrogen receptor (ER)−, progesterone receptor (PgR)−, and MIB1-positive cells according to immunohistochemistry (IHC); human epidermal growth factor receptor 2 (*HER2*) expression status; and relapse status. Patients were subdivided into molecular phenotypes using IHC surrogates (luminal A [ER−/PR+/HER2−], luminal B [ER+/PR+/HER2+ or ER+/PR+/HER2−/MIB1 index high], HER2 [in figure, shown as H; ER−/PR−/HER2+], and TN [ER−/PR−/HER2−]). Clinical risk estimated by age and *HER2* expression status is shown. **b** *GATA3* and *PIK3CA* mutations are shown (red and blue, respectively). Only the results for nonsynonymous mutations are presented. In total, 40 of 72 (56%) patients carried nonsynonymous *GATA3* mutations. Nonsynonymous *PIK3CA* mutations were detected in 36 of 72 (50%) patients. **c** The genes that overlapped among patients in the discovery cohort are shown. *1: *GATA3* mutation-positive ductal carcinoma in situ (DCIS). *2: *GATA3* mutation-negative, ER-positive DCIS with low PgR expression. *3: *PIK3CA* mutation-positive DCIS that does not satisfy *1 or *2.

Targeted sequencing was conducted at a sufficient sequencing depth (averaging ×592.2). The obtained mutation graph is shown in Fig. 2. In total, 40 (56%) and 36 patients (50%) harbored *GATA3* and *PIK3CA* mutations, respectively. In accordance with the results for cohort 2, *GATA3* mutations were positively associated with relapse (OR = 7.8; 95% CI = 1.17–88.4), whereas *PIK3CA* mutations tended to be negatively associated with relapse (OR = 0.45; 95% CI = 0.12–1.7). Of the 180 genes tested, none were superior to these two factors as a predictive marker. For PgR protein expression, low PgR expression was again correlated with relapse in cohort 3 (OR = 25.6; 95% CI = 3.64–142.2). In conjunction with cohort 2, we identified *GATA3* mutations in 8 out of 11 cases for which PgR was low despite ER positivity. Considering these findings, we believe that the results from the discovery cohort reflect the true nature of DCIS.

### Analyses of patients with DCIS for molecular dissection

**Fig. 3:**
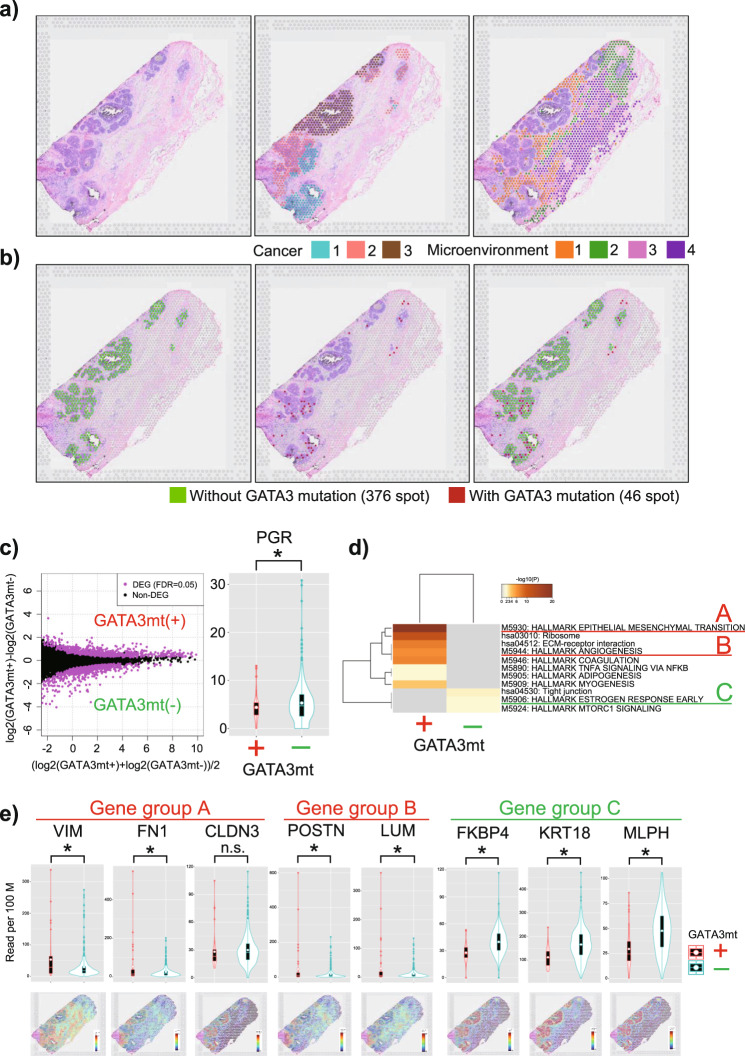
Spatial transcriptome analysis of a patient with *GATA3* mutation-positive ductal carcinoma in situ (DCIS) (Patient A). **a** Visualization of the Visium results for Patient A. Hematoxylin and eosin staining (left). In total, 2043 spots in the tissue are represented. Morphologically, those cells were classified as cancer cells in situ (405 spots) and noncancer cells (1638 spots). Three cancer cell populations classified via nonhierarchical k-means clustering (k = 9) are shown with different colors (middle panel). Four noncancer spots are similarly shown to represent the gene expression reflecting the microenvironments at the corresponding positions (right panel). **b** The spots in which *GATA3* mutations were detected as transcriptomic tags are colored red. Of the 422 spots morphologically located with cancer cells, a *GATA3* mutation read was found for 46 spots. **c** MA plot presenting differentially expressed genes (DEGs) between spots with and without *GATA3* mutations. In total, 1468 DEGs were detected at a false discovery rate of <0.05 (left). The right panel shows a comparison of progesterone receptor (*PgR*) expression at each spot. Decreased *PgR* expression was more frequently observed in the spots with *GATA3* mutations (*P* = 0.02901, one-sided *t*-test). **d** Results of the enrichment test for the MSigDB v5.1 Hallmark gene set collection (HALLMARK) and Kyoto Encyclopedia of Genes and Genomes (KEGG) pathways are presented. Each band represents one enriched term or pathway. The level of enrichment is color-coded as the −log 10 *p* value. As indicated, in the spots with *GATA3* mutations, the DEGs were mainly enriched in epithelial-mesenchymal transition (“Gene group A”) and angiogenesis (“Gene group B”). Conversely, in the spots without *GATA3* mutations, the DEGs were mainly enriched in tight junctions, estrogen response (“Gene group C”), and MTORC1 signaling. **e** Violin plots showing the expression of representative genes corresponding to the gene groups of A–C. The expression of the indicated gene was compared between the spots with and without *GATA3* mutations in the upper panel. *P < 0.05 (one-sided), n.s.: not significant. The gene expression levels in the tissue are shown in the bottom panel.

To determine the molecular etiology underlying the high-risk markers identified, we precisely investigated three additional representative patients with fresh frozen specimens using STseq. Patient A had DCIS with a *GATA3* mutation (Fig. 2, *1) and microinvasion, and the lesion was regarded as true DCIS. Patient B had DCIS with low PgR expression (Fig. 2, *2) and microinvasion, and the lesion was regarded as true DCIS. Patient C had DCIS with a *PIK3CA* mutation (Fig. 2, *3) without any microinvasion, and the lesion was regarded as possibly false DCIS (low-risk DCIS). The clinicopathological information for each patient is provided in Supplementary Table S4.

### STseq of a DCIS lesion harboring a GATA3 mutation

First, we profiled the spatial gene expression of the specimen from Patient A via STseq using the Visium platform of 10X Genomics (Pleasanton, CA, USA), which is a recently developed barcoding-based spatial transcriptomics technology. This patient was diagnosed with DCIS in the preoperative pathological diagnosis but was found to have a microinvasion site in the postoperative pathological diagnosis. Thus, this DCIS lesion was identified as a true precursor for IDC. The panel sequencing analysis at an average depth of ×2391.4 revealed that this sample harbored a *GATA3* mutation (exon 4: c.865dupG: p. C288fs, variant allele frequency [VAF] = 7%; the full mutation list is provided in Supplementary Data 3). Interestingly, a *PIK3CA* mutation was also detected at a higher VAF (exon 5:c.T1035A:p. N345K, VAF = 26.1%), suggesting that the emerging *GATA3* mutation was overwriting the basal features of DCIS with the *PIK3CA* mutation.

For the Visium analysis, we collected 799,133,428 sequencing reads at a sequencing saturation of 86.2%. The number of analyzed spots (55 µm in diameter) was 2043, which contained a median of 7469 unique molecular identifier (UMI) reads and a median of 2928 detected genes per spot (Supplementary Data 4). As shown in the middle panel of Fig. 3a, cancer cell spots were classified into three groups via nonhierarchical k-means clustering (k = 9), suggesting the presence of heterogeneity in the DCIS lesion. Additionally, noncancer spots, which may represent the microenvironment surrounding the cancer cells, were classified into four clusters. Among a total of 422 spots representing pathologically identified cancer cells, *GATA3* mutation reads were detected in 46 spots by analyzing Visium reads (Fig. 3b, Supplementary Fig. S6). Fortunately, in this patient, the *GATA3* mutation was located at the 3′-end of the transcript and could thus be represented by the Visium reads. To investigate whether cells harboring the *GATA3* mutation (spots) are clonal, we examined the *GATA3* mutation sites on Visium reads of 46 spots carrying *GATA3* mutations. The mutations were present exactly at the same site (exon 4: c.865dupG: p. C288fs) in all 46 spots, suggesting that they are of monoclonal origin. Using the TCC R package^27^, we detected differentially expressed genes (DEGs) between the spots with and without *GATA3* mutations. In total, 1468 DEGs were detected (false discovery rate [FDR] < 0.05, Fig. 3c, left panel). As shown in Fig. 1d, downregulated PgR expression was noted in the spots with *GATA3* mutations (*P* = 0.02901; *t*-test; Fig. 3c, right panel). To predict the functional consequence of the detected DEGs, we conducted pathway analyses using Metascape (http://metascape.org/)^28^. Nine key pathways, which were associated with 11 cancer hallmarks, were affected in the cancer spots with *GATA3* mutations compared with the findings in the mutation-negative spots (Fig. 3d, Supplementary Data 5). In particular, the key genes and pivotal pathways included EMT and angiogenesis. Importantly, the key genes with expression changes were those identified to occur in response to aberrant *GATA3* function, such as *VIM* and *FN1* (Fig. 3e). These results indicate that *GATA3* mutations arise during DCIS progression accompanied by malignant features. It has been reported that increased VIM expression occurs downstream of *GATA3* mutations in luminal cancer cells^24^; thus, the observed *GATA3* mutations in the spots could represent prior genetic alterations during malignant development. Conversely, in the spots without *GATA3* mutations, the DEGs were mainly enriched in estrogen response, tight junction, and mTORC1 signaling pathways, suggesting that the cells in those spots acquired the minimum changes permitting cell transformation.

### STseq of a DCIS lesion with low PgR expression but no GATA3 mutation

We next investigated Patient B as an exceptional patient with low PgR expression despite the absence of *GATA3* mutation (Fig. 2, *2). From targeted sequencing at an average depth of ×3030.6, a *PIK3CA* mutation was detected (exon 21:c.A3140T:p. H1047L, VAF = 40%, Supplementary Data 3), whereas no *GATA3* mutation was detected even at this sufficient sequencing depth. Therefore, this patient was expected to have a good prognosis on a molecular basis. Nevertheless, postoperative pathological examinations revealed microinvasion, indicating that this DCIS lesion was a true precursor of IDC. Immunohistochemical analysis illustrated that this patient was ER-positive and PgR-negative.

**Fig. 4:**
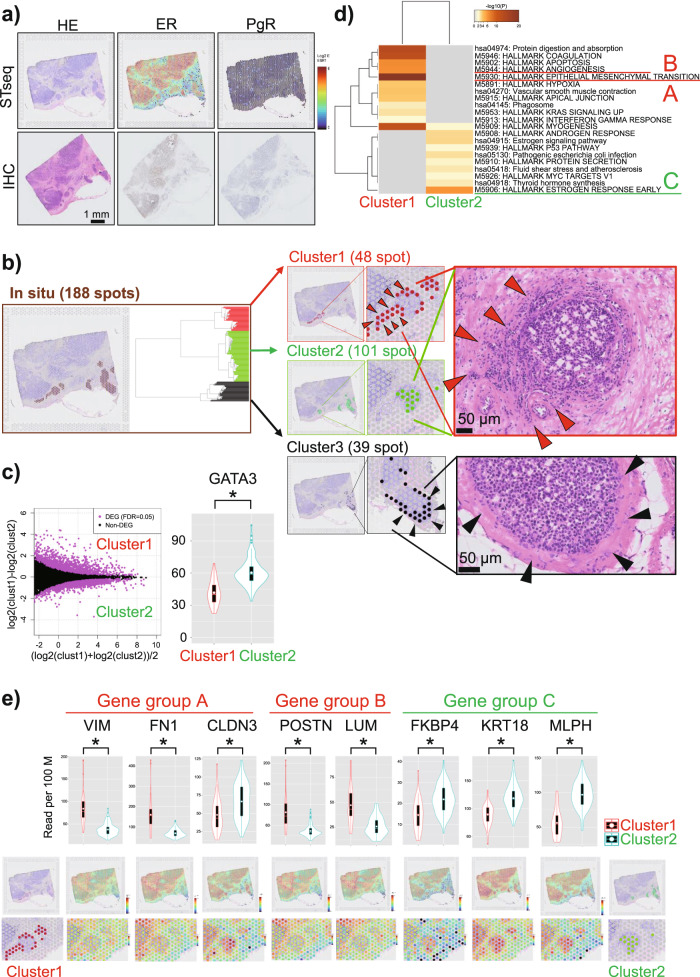
Spatial transcriptome analysis of low progesterone receptor (PgR) expression in a patient with estrogen receptor (ER)-positive ductal carcinoma in situ (DCIS) (Patient B). **a** Visualization of the Visium results for Patient B. The upper row shows the expression of genes indicated in the top margin. The lower panels show the results of hematoxylin and eosin staining and immunostaining for ER and PgR. **b** Dendrogram generated from hierarchical clustering performed across 188 spots indicated as colored dots (left panel). The middle panel presents the three distinct clusters detected via hierarchical clustering. The right panel shows the originating positions of the indicated spots in the tissue. The rightmost panels present the following observations. Namely, the 48 spots of cluster 1 overlapped with the locations of DCIS cells that were about to invade (red arrow) on the basis of their morphology. The spots of cluster 2 were located in ducts that were anatomically in the same position as those of cluster 1, but their locations coincided with those of DCIS cells that had not invaded. The location of cluster 3 was consistent with that of DCIS cells that were not invasive (black arrow) on the basis of their morphology. **c** MA plot showing differentially expressed genes (DEGs) between clusters 1 and 2. In total, 2747 DEGs were detected (false discovery rate <0.05, left). The right panel shows a comparison of *GATA3* expression (*P* < 0.001, one-sided *t*-test). Decreased *GATA3* expression was observed in cluster 1. **d** MSigDB v5.1 Hallmark gene set collection (HALLMARK) and Kyoto Encyclopedia of Genes and Genomes (KEGG) pathway analyses were conducted using the selected DEGs (the top 100 genes were used) and the Metascape tool. Each band represents one enriched term or pathway colored according to the −log 10 *p* value. In spots in cluster 1, the DEGs were mainly enriched in epithelial-mesenchymal transition (“Gene group A”) and angiogenesis (“Gene group B”). Conversely, in spots in cluster 2, the DEGs were mainly enriched in the estrogen response (“Gene group C”). **e** Violin plots showing the expression of each gene in A, B, and C in spots from clusters 1 and 2 (upper panel). Visualization of the expression of each gene on the Visium slide (lower panel). *P < 0.05 (one-sided), n.s.: not significant.

Visium analysis uncovered heterogeneous gene expression depending on the location of cells in both cancerous and noncancerous regions (Supplementary Fig. S7). PgR expression was downregulated despite the observed high ER expression (Fig. 4a, upper panel), consistent with the immunostaining findings (Fig. 4a, lower panel). Focusing on the changes in gene expression in DCIS cells, 188 spots that were morphologically located in the intraductal regions were manually selected. Unsupervised hierarchical clustering of these spots identified three apparent clusters (Fig. 4b, left panel). Spatially, the different clusters corresponded to different regions (Fig. 4b, middle panel). Importantly, the 48 spots of cluster 1 (colored red) overlapped with the location of DCIS cells that were about to invade on the basis of their morphology (indicated by the red arrowhead in the right panel of Fig. 4b). Meanwhile, the 101 spots of cluster 2 (colored green) were located in the center of the same duct and were regarded as cells that were not invading the stroma anatomically (indicated by green spots in the middle panel of Fig. 4b). Differential expression analysis between clusters 1 and 2 identified 2747 DEGs (FDR < 0.05, Fig. 4c, left). Interestingly, *GATA3* expression was significantly downregulated in cluster 1 (Fig. 4c, right). Functional aberration of *GATA3* likely occurred at the gene level in this patient despite the absence of a mutation of this gene. Consistently, *GATA3*-centered gene expression changes were observed in this patient as judged by the results of gene enrichment and pathway analyses, as observed for Patient A (Fig. 4d, e). A total of 21 pathways and 18 cancer hallmarks, including EMT and angiogenesis pathways, were affected (Supplementary Data 5). As observed for Patient A, in cluster 2, which had not invaded the stroma, the DEGs mainly represented estrogen response-related genes. Collectively, in this patient as well, we concluded that *GATA3* plays pivotal roles via changes in its expression. Even without genomic mutations, aberrant *GATA3* expression may result in an equivalent consequence in some patients.

### Integrated STseq and scDNA-seq analysis of a patient with DCIS and PIK3CA mutations

**Fig. 5:**
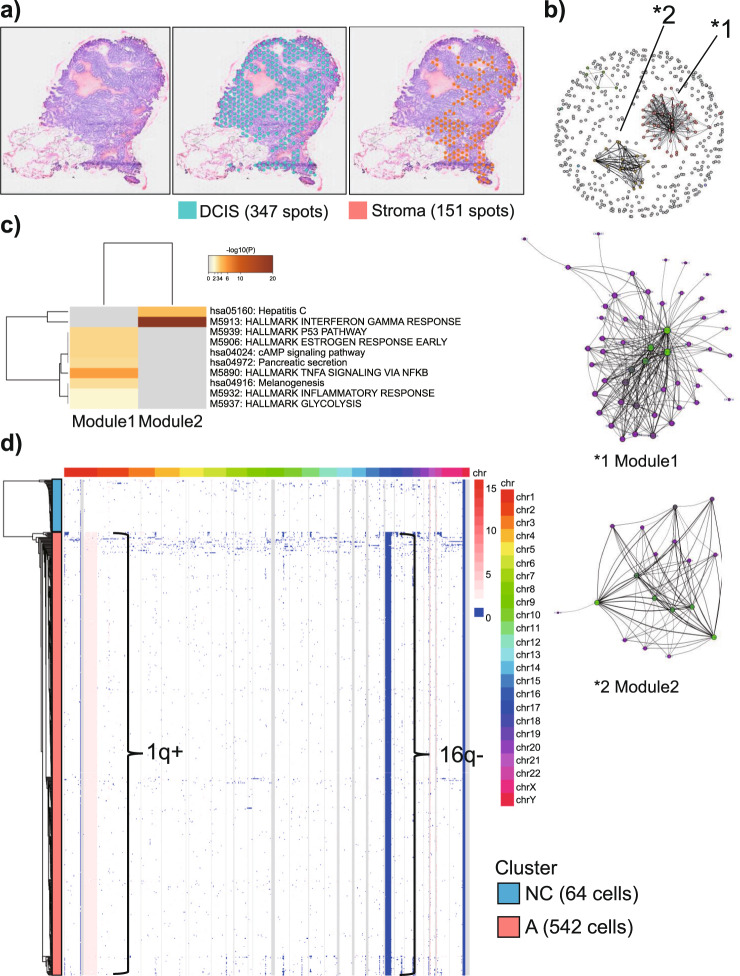
Integrated spatial transcriptome and single-cell DNA sequencing analysis of a patient with *PIK3CA* mutation-positive ductal carcinoma in situ (DCIS) (Patient C). **a** Visualization of the Visium results for Patient C. Hematoxylin and eosin staining (left). The number of spots in the tissue was 594. Spots were classified into two clusters via unhierarchical k-means clustering. The clusters almost completely overlapped with morphologically identified cancer cells (middle panel) and noncancer cells (stromal cells, right panel). **b** Coexpression network analysis of transcriptomes in DCIS spots. Weighted gene coexpression network analysis was applied to build the coexpression network and identify gene modules. The nodes and edges indicate genes and significant correlations between genes, respectively. In the top panel, the two nodes that appeared to have changed from normal cells are indicated by asterisks. Middle and lower panels present the subnetworks in modules (*1) and (*2), respectively. Node centrality, defined as the sum of strength, is represented by node size and color. **c** Pathway analysis was performed for each module using the Metascape tool. Each band represents one enriched term or pathway colored according to the −log 10 *p* value. Pathway enrichment analysis revealed that genes involved in p53 and estrogen signaling pathways were enriched in module 1, which shares the characteristics observed for the benign-appearing spots in Patients A and B, indicating that malignant transition had not occurred in this cancer. **d** Heatmap showing copy number variation at the single-cell level. The color scale for the copy number changes is shown at the right margin. The corresponding chromosomal locations are also shown. A total of 594 cells are represented. Clustering was performed as described in the “Methods”. Single-cell copy number variation analysis revealed two clusters, namely, clusters A and NC (noncancer). Cluster A features 1q+ and 16q− structural variants and represents DCIS cells. Because cluster NC does not have a structural variant, it was considered to represent noncancer cells from Patient C.

Last, we analyzed the case of Patient C as a possibly false DCIS case that was unlikely to progress to IDC (low-risk DCIS). This patient belonged to the group indicated as *3 in Fig. 2 in cohort 3, which was characterized by *PIK3CA* mutations, an absence of *GATA3* mutations or downregulation, no microinvasion, and no relapse after surgery. The DCIS lesion in this patient harbored two *PIK3CA* mutations (exon 10:c.G1633A:p. E545K, exon10:c.A1634G:p. E545G, VAF = 25% for both mutations), as determined via whole-exome sequencing with an average depth of ×134.8. The Visium analysis revealed a monotonous gene expression pattern, in line with the morphologically monoclonal structure of this cancer (Fig. 5a, left panel). Indeed, the spots were roughly separated into two clusters via nonhierarchical clustering (Fig. 5a, middle and right panels). They almost completely overlapped with the morphologically determined cancer and noncancer cells (stromal cells). Thus, we compared 347 spots in the cancer cells (blue spots) with 151 spots in the stromal cells (orange spots) using DEG analysis and identified 508 upregulated DEGs in the cancer spots (FDR < 0.05). Weighted gene coexpression network analysis (WGCNA) revealed that only two major nodes (modules) were altered in the cancer spots compared with those in the noncancer spots (Fig. 5b, upper panel, Supplementary Data 6). We conducted subnetwork analysis to identify the hub genes (Fig. 5b, middle and lower panels). Pathway enrichment analysis of the hub genes revealed that genes involved in the estrogen response and p53 pathways were enriched in module 1 (Fig. 5c, Supplementary Data 5), which shares the characteristics observed for the benign-appearing spots in Patients A and B, indicating that malignant transition had not occurred in this lesion.

To further examine the genomic clonality of this cancer, we performed scDNA analysis. Cells in the cancer specimen were grouped into two clusters. Cluster A (542 cells), as shown in Fig. 5d, contained genomic aberrations at 1q+ and 16q−. These mutations, namely, a gain of 1q and loss of 16q, are frequently observed in sporadic breast cancers^29,30^, especially histologically low-grade DCIS^31,32^, supporting the presumption that the lesion in Patient C was low-grade DCIS. Conversely, cluster NC (64 cells) had no genomic aberrations, and thus, these cells were considered to be noncancerous cells. The proportions of cancerous and noncancerous cells based on the morphological information (approximately 90 and 10%, respectively) were consistent with those observed via scDNA analysis (cluster A, 89%; cluster NC, 11%). Significantly, no subclone was detected in cluster A, indicating the absence of additional diversification of cancer cells. These results differed from those for a malignant lesion (Patient 7 and 10, who were also subjected to scDNA analysis, Supplementary Figs. S8 and S9).

Overall, the DCIS lesion in Patient C did not harbor any genomic or transcriptomic alterations leading to malignant transition, such as EMT and angiogenesis, which were observed in patients with *GATA3* mutation or downregulation, supporting the presumption that the lesion was possibly false DCIS. From the aforementioned clinical and molecular analyses, we propose the following five critical markers for a new DCIS classification approach: age <45 years, *HER2* amplification, *GATA3* mutation or downregulation, PgR protein negativity (for high-risk and true DCIS), and *PIK3CA* mutation positivity (for false DCIS).

## Discussion

In this study, we identified *GATA3* mutation as a potential marker for classifying DCIS. *PIK3CA* mutation negativity and PgR protein negativity in patients with ER-positive DCIS were also suggested to be risk factors. We showed that conventional clinicopathological factors are also critical for predicting the risk of IDC development, including age (≥45 years vs. <45 years) and *HER2* status (negative vs. positive). *HER2* amplification is a well-known poor prognostic factor in IDC in the absence of HER2-targeted therapy. However, the frequency of *HER2* amplification differs between DCIS and IDC^33–37^, and whether *HER2* amplification is a risk factor for DCIS remains disputable^38^. The HR for HER2 positivity/age ≥45 years versus HER2 negativity/age ≥45 years was 1.54 (95% CI = 0.31–7.65, *P* = 0.595). The HR for HER2 negativity/age <45 years versus HER2 negativity/age ≥45 was 2.43 (95% CI = 0.78–7.52, *P* = 0.125). In contrast, the HR for HER2 positivity/age <45 years versus HER2 negativity/age ≥45 was 11.03 (95% CI = 3.54–34.4, *P* < 0.0001). Although the *P* value of the interaction test was 0.121, these results may suggest an interaction between age and HER2 amplification.

To improve the prediction accuracy, we found that *GATA3* mutational information is particularly useful. A limited number of studies have investigated *GATA3* mutations in DCIS^39^, and the prognostic consequences of mutations remain to be clarified^40,41^. *GATA3* promotes differentiation of luminal cells and inhibits infiltration and metastasis of breast cancer cells^42–46^. *GATA3* mutation, which occurs at a high incidence rate in IDC^47^, is believed to abolish this critical function and promote the progression of cells to a more advanced stage^48^. Therefore, *GATA3* mutations may play critical roles in migration and invasion of cancer cells but not in initial carcinogenesis. In clinical practice, examination of *GATA3* mutations is not always possible. In such patients, our results suggest that PgR protein expression is associated with *GATA3* mutation. Routine measurement of PgR protein expression would generally predict the functionality of GATA3 in patients with ER-positive DCIS. A future extensive analysis is needed to elucidate how PgR negativity is caused by *GATA3* mutation. *PIK3CA* mutations are reported to be crucial for breast carcinogenesis^49,50^. However, *PIK3CA* mutation is correlated with a favorable prognosis in IDC^26^. It was also recently reported that mutations in the *PIK3CA* kinase domain and the absence of copy number gains in DCIS protect against progression to invasive cancer^51^. Our results indicating that DCIS cells with *PIK3CA* mutations possess a monoclonal structure in which expression modules are required specifically for proliferation pathways support previous findings and suggest that *PIK3CA* mutations alone should be included as a marker for predicting a lower risk of progression to IDC and for reassessment of treatment options. A logical interpretation is that sequential mutation of *GATA3* facilitates acquisition of invasive characteristics following *PIK3CA* mutations to promote tumor initiation.

In this study, using spatial transcriptome analysis, we directly demonstrated that impaired function of *GATA3* in DCIS causes progression to invasive cancer in several cases. In fact, the new STseq method provided preliminary but important clues. To show the variation in spatial performance, we extracted 422 spots consistent with cancer cells using Visium and compared the number of reads between 46 spots with and 376 spots without *GATA3* mutant reads. The results showed that there were no differences in the number of reads between the two groups (Supplemental Fig. S10a). Furthermore, 142 spots with reads at the *GATA3* mutation site were extracted, and the distribution of the number of reads was evaluated (Supplementary Fig. S10b). The results demonstrated that there were no mutant reads, although the distribution of reads in the 96 spots without mutant reads was similar to that in the 46 spots with mutant reads. Thus, spatial ascertainment bias appeared to have almost no impact. Additionally, the STseq method may be less accurate regarding cell resolution than imaging mass cytometry (IMC). However, it should be noted that the data that we obtained contained more comprehensive information on spatial expression than IMC. In this regard, we consider it a more suitable method for exploratory research not limited to genes. Another obvious limitation of this study was that only a limited number of samples were used. In our cohort, despite being a relatively large cohort among studies on DCIS, only 20 patients (4.6%) among 431 patients experienced relapse. Additionally, the prognostic observation period for breast cancer is usually 10 years, which is longer than that for other cancer types. Therefore, at this point, it is impossible to verify future recurrence in cases where recurrence was not confirmed. We have no choice but to say that each association is low. To complement this, by performing spatial transcriptome analysis of DCIS using STseq for three representative cases, we directly demonstrated that *GATA3* mutation-harboring DCIS cells become more aggressive. We also demonstrated that DCIS cells with PIK3CA mutations did not harbor any genomic or transcriptomic alterations leading to malignant transition. We plan to continue follow-up of the disease going forward. In the future, clinical studies of DCIS observing its natural course in the absence of treatment, including ongoing trials^12–14^, may provide an accurate algorithm for distinguishing between high- and low-risk DCIS using markers including those proposed in this study, thereby avoiding unnecessary treatment.

## Methods

### Ethics approval

This study was approved (approval number: 2297-i103) by the Clinical Ethics Committee of St. Marianna University, and a waiver of consent was granted for the use of archival clinical samples from the Department of Pathology.

### Clinicopathological data

Clinicopathological data were obtained from 431 consecutive patients with DCIS who underwent surgery at the University Hospital of St. Marianna University School of Medicine between 2007 and 2012. The detailed clinicopathological data are provided in Supplementary Table S1 and Supplementary Data 1.

### Exome library construction and whole-exome sequencing

For the discovery cohort, we selected formalin-fixed, paraffin-embedded (FFPE) tissue samples from 21 patients with true DCIS (primary tumor) and four patients with IDC (relapsed tumors). In each patient, epithelial areas in DCIS and normal epithelial tissue were identified via hematoxylin and eosin staining of the corresponding cryosections. Epithelial areas of interest were confirmed via histological assessment of each patient by two histopathologists. Microdissected tissues (10-μm thick) were obtained using the Roche Automated Tissue Dissection System (Roche) and used to selectively isolate DCIS and nontumor cells for whole-exome sequencing analysis, reducing the extent of contamination by stromal cells (Supplementary Fig. S2). All the genomic analyses of lesions in this study were from only one lesion from each patient and not from pooled lesions collected from multiple sites. DNA from the microdissected tissues was extracted using QIAamp DNA Mini and Micro kits (Qiagen, Crawley, UK). DNA quality was assessed using a Tape Station (Agilent Technologies), and the concentration was assessed using a Bioanalyzer 2100 (Agilent Technologies).

Next-generation sequencing DNA libraries were prepared for whole-exome sequencing using 100–200 ng of DNA. In brief, whole-exome libraries were prepared using a SureSelect XT HS and XT Low Input Target Enrichment Kit (Agilent, UK) and OneSeq SS 300 kb Backbone + Human All Exon V7 capture library (Agilent Technology) following the manufacturer’s guidelines. Whole-exome libraries were sequenced using 100-bp paired-end runs on an Illumina HiSeq 2500/3000 system (Illumina).

The sequencing data were analyzed using a custom pipeline. In brief, sequencing reads were aligned to the human genome (hg 19) using Burrows-Wheeler Aligner Mem (version 0.7.17)^52^. Duplications were marked using Picard Tools version 2.18.25 (http://broadinstitute.github.io/picard). Insertion–deletion realignment and base recalibration were achieved using GATK version 4.0.12-0^53^.

Somatic variants were detected using an ensemble approach with two variant callers: MuTect2^54^ and Genomon pipeline^55^. Variant annotation was performed using ANNOVAR^56^. To obtain the final set of mutation calls, we used a two-step approach: (1) to reduce false-positive calls, the mutant variant frequency must be at least 4% of total reads; (2) to remove any spurious variant calls arising as a consequence of sequencing artifacts, we checked bam reads using Integrative Genomics Viewer (http://www.broadinstitute.org/igv/). Only variants with the following functional classification were considered in this study: nonsynonymous single nucleotide variants, stop gain mutations, and frameshift mutations. Full mutation list is provided in Supplementary Data 7.

### Target sequencing library construction and deep targeted sequencing

For the validation study, we conducted deep targeted sequencing. We selected 72 patients with true DCIS and available FFPE tissue. Because of the limited number of specimens, only tumor samples were analyzed. Manually macrodissected tissues were used to enrich DCIS cells. DNA from the macrodissected tissues was extracted using QIAamp DNA Mini and Micro kits and tested for quality using Tape Station (Agilent Technologies), and the concentration was assessed using a Bioanalyzer 2100.

Next-generation sequencing DNA libraries were prepared for target sequencing using 100–200 ng of DNA. In brief, target sequence libraries were prepared using a SureSelect XT HS and XT Low Input Target Enrichment Kit and custom sequencing panel following the manufacturer’s instructions. A custom sequencing panel containing 180 genes was designed (full list in Supplementary Table S3). This panel targeted genes that were duplicated in the whole-exome sequencing study of the preceding 21 patients, including *GATA3*, *PIK3CA*, and breast cancer-related genes. The total region size of the custom panel was 1.5 Mbp. Target sequence libraries were sequenced using 100-bp paired-end runs on the Illumina NovaSeq platform. The sequencing data were analyzed using a custom pipeline as described in the “Exome library construction and whole-exome sequencing” subsection. Full mutation list is provided in Supplementary Data 8.

### Subclonal analysis

The number of subclones contributing to each sample was estimated using the PyClone program version 0.13^23^. We used the Sequenza package to define the CNV from whole-exome sequencing data^57^. We set the major and minor allele copy numbers to those obtained from Sequenza, allowing clustering to simply group clonal and subclonal mutations. PyClone was operated with 10,000 iterations and default parameters. PyClone results were analyzed using CloneEvol to infer and visualize clonal evolution as described previously^23^.

### STseq using Visium

We selected three patients for STseq using Visium. The clinical information for each patient is provided in Supplementary Table 4.

Each sample was collected immediately after surgical removal and embedded in optimal cutting temperature (OCT) compound (TissueTek Sakura) in a 10 mm × 10 mm cryomold at −80 °C until use. Frozen samples embedded in OCT compound were sectioned at a thickness of 10 μm (Leica CM3050 S). Libraries for Visium were prepared according to the Visium Spatial Gene Expression User Guide (CG000239_VisiumSpatialGeneExpression_UserGuide_Rev_A.pdf). Tissue was permeabilized for 6 min, which was identified as the optimal time in tissue optimization time course experiments.

Libraries were sequenced on a NovaSeq 6000 System (Illumina) using a NovaSeq S4 Reagent Kit (200 cycles, 20027466, Illumina) at a sufficient sequencing depth (approximately 700 million to 1.2 billion reads per sample).

Sequencing was performed using the following read protocol: read 1, 28 cycles; i7 index read, 10 cycles; i5 index read, 10 cycles; read 2, 91 cycles. Raw FASTQ files and histology images were processed using Space Ranger software v1.0.0 (https://support.10xgenomics.com/spatial-gene-expression/software/pipelines/latest/installation). To visualize spatial expression using histological images, the raw Visium files for each sample were read into Loupe Browser software v4.0.0 (https://support.10xgenomics.com/spatial-gene-expression/software/downloads/latest).

To assign individual spots to tumor cells or cells that compose the microenvironment, we compared the clusters morphologically annotated by a pathologist to data-driven spatial clusters using k-means clustering results provided by 10× Genomics Space Ranger software (Figs. 3a,  5a, Supplementary Fig. S7).

We extracted reads with confirmed *GATA3* mutations in whole-exome sequencing from the Visium alignment results and identified spots with *GATA3* mutations (Fig. 3b, Supplementary Fig. S6).

### Differential expression analysis and pathway analysis of each Visium patient

We performed differential expression analysis and pathway analyses at the spot level using our Visium data.

In Patient A, we compared 46 spots with *GATA3* mutations (red in Fig. 3b) to 376 spots without *GATA3* mutations (green in Fig. 3b). Count data in raw Visium files were used. DEGs were identified using the TCC package^27^ and a filtering threshold of FDR < 0.01. The TCC package was also used to generate an MA plot to visualize DEGs. Metascape^28^ (http://metascape.org) was used to perform pathway enrichment analysis. The top 100 genes according to the FDR were subjected to Metascape, which was performed on two groups of gene sets, namely, the MSigDB version 5.1 Hallmark gene set collection (HALLMARK)^58^ and Kyoto Encyclopedia of Genes and Genomes (KEGG). Pathways were considered statistically significant at *P* ≤ 0.05. To assess whether there were differences in mRNA expression between the spots with and without *GATA3* mutations using an unpaired *t*-test (Fig. 3e), row count data were normalized as counts per 10,000 by dividing each spatial spot column by the sum of its counts and multiplying by 10,000.

In Patient B, to focus on gene expression changes in DCIS cells, we manually selected 188 spots morphologically located in the region of the milk ducts. Unsupervised hierarchical clustering based on Spearman’s distance and Ward’s linkage was used to construct a tree relating the clusters. Hierarchical clustering analysis of these spots identified three apparent clusters (clusters 1–3). The 48 spots of cluster 1 overlapped with the location of DCIS cells that were about to invade on the basis of their morphology. Meanwhile, the 101 spots of cluster 2 were located in the proximal ducts that were anatomically the same region as the spots of cluster 1. We compared the 48 spots in cluster 1 (red in Fig. 4b) to the 101 spots in cluster 2 (green in Fig. 4b). Count data in raw Visium files were used. DEGs were identified using the TCC package and a filtering threshold of FDR < 0.01. The TCC package was also used to generate an MA plot to visualize DEGs. Metascape was used to perform pathway enrichment analysis using the HALLMARK and KEGG gene sets. Pathways were considered statistically significant at *P* ≤ 0.05. To assess differences in mRNA expression between the spots in clusters 1 and 2 using an unpaired *t*-test (Fig. 4e), row count data were normalized as counts per 10,000 by dividing each spatial spot column by the sum of its counts and multiplying by 10,000.

### Coexpression network analysis of Visium Patient C

Patient C exhibited a monotonous gene expression pattern, which was consistent with the morphologically monoclonal structure of this cancer (Fig. 5a). The spots were roughly separated into two clusters via nonhierarchical k-means clustering. They almost completely overlapped with morphological cancer (347 spots) and noncancer cells (151 spots). Thus, we compared the 347 spots with cancer cells (blue in Fig. 5a) with the 151 spots with stromal cells (orange in Fig. 5b). DEG analysis using the TCC package (as described in the differential expression analysis and pathway analysis of each Visium patient subsection) between the cancer and noncancer spots revealed 2364 DEGs (Supplementary Data 6).

To group related genes into gene modules (clusters) based on their coexpression patterns, we used WGCNA^59^. For WGCNA, 508 DEGs that were upregulated in cancer spots were used to construct a coexpression network. Using the pickSoftThreshold function with a fit value exceeding RsquaredCut, which was 0.8, the power (β) parameter was inferred to be 3. WGCNA could not confidently assign 439 genes to any of the modules because they displayed little correlation with any other gene. These uncorrelated genes were designated module 0 and excluded from the rest of the analysis. We conducted subnetwork analysis to identify the hub genes in each module. Pathway enrichment analysis of these hub genes using Metascape revealed that genes involved in the estrogen response and p53 pathways were enriched in module 1.

### scDNA library preparation and scDNA-seq

Tissue biopsies were obtained from surgically resected primary DCIS samples. Samples were washed in PBS (Wako 045-29795), mechanically dissociated using a razor blade, and digested in DMEM (Wako 041-29775) containing collagenase Type P, 2 mg/mL (Roche 11213857001). Cellular debris and aggregates were filtered using a 40-μm cell strainer (CosmoBio) prior to scDNA-seq. Single-cell suspensions were loaded into a Chromium 10× device according to the standard protocol provided with a Chromium Single-Cell DNA Reagent Kit (10× Genomics).

For droplet-enabled scDNA-seq, we used the 100-bp paired-end Illumina HiSeq3000. Sequencing data were processed using cellranger-dna-1.1.0 (refdata-GRCh38-1.0.0.), which automated sample demultiplexing, read alignment by bwa mem with -M options, correction of GC bias by fitting to the quadratic function that minimizes the entropy of the read count histogram per 20 kbp, CNV calling with a 20-kb bin size, and report generation. We excluded noisy cells and cells with ploidy <1.9 or >2.0 (Her2 > 8 is an exception) from the cellranger-dna results.

A CNV heatmap was plotted using the pheatmap function in the R package, and using the Manhattan distance, we computed hierarchical clustering using Ward’s minimum variance method. To identify subclusters from CNA Data, the optimal “k” (number of clusters) was determined using the elbow Method and Silhouette Method in the R package ‘factoextra,’ viz_nbclust function (FUN = hcut, k.max = 15). The tree was cut into k clusters to determine the number of clones and optimized manually.

### Immunohistochemistry and measurement of protein expression

Paraffin tissue sections 4-μm thick on coated slides were deparaffinized using routine techniques. ER, PR, and HER2 expression was determined using standard immunohistochemical and fluorescent in situ hybridization techniques.

HER2 overexpression was analyzed according to the American Society of Clinical Oncology and College of American Pathologists 2013 recommendations; specifically, HER2 staining was considered positive if the circumferential membrane staining was complete, intense, and present in more than 10% of tumor cells (HER2 score 3+) or if circumferential membrane staining was incomplete and/or weak/moderate and present in more than 10% of tumor cells (HER2 score 2+) if HER2 overexpression could be confirmed via fluorescence in situ hybridization. HER2 staining was considered negative when incomplete membrane staining was faint/barely visible in >10% of the tumor cells (HER2 score 1+) or when no staining was observed (HER2 score 0)^60^.

### Statistical analyses

Statistical analyses were performed using GraphPad Prism version 8.0 and R version 3.5.0. Fisher’s exact tests were employed for comparisons of unordered categorical variables. Student’s *t*-test was used to compare continuous variables and ordered categorical variables.

Relapse-free survival was defined as the time from the date of surgery to that of relapse or the last contact. Survival curves were constructed using the Kaplan–Meier method and compared using a log-rank test. Cox proportional hazard regression models, including unadjusted models and models adjusted for available prognostic clinical and genomic covariates, were constructed to calculate HRs and 95% CIs. Statistical significance was accepted at *P* < 0.05 (two-sided, unless otherwise indicated).

### Reporting summary

Further information on research design is available in the Nature Research Reporting Summary linked to this article.

## Supplementary information

Supplementary Information

Description of Additional Supplementary Files

Supplementary Data 1

Supplementary Data 2

Supplementary Data 3

Supplementary Data 4

Supplementary Data 5

Supplementary Data 6

Supplementary Data 7

Supplementary Data 8

Reporting Summary

## Data Availability

All sequencing data and pathological images for STseq have been deposited in the DNA Data Bank of Japan under accession number JGAS00000000202. Any other data associated with this study are available upon reasonable request.

## References

[1] Fechner RE (1993). One century of mammary carcinoma in situ: what have we learned?. Am. J. Clin. Pathol..

[2] Polyak K (2008). Is breast tumor progression really linear?. Clin. Cancer Res..

[3] Gorringe KL, Fox SB (2017). Ductal carcinoma in situ biology, biomarkers, and diagnosis. Front. Oncol..

[4] Kerlikowske K (2010). Epidemiology of ductal carcinoma in situ. J. Natl Cancer Inst. Monogr..

[5] Kuerer HM (2009). Ductal carcinoma in situ: state of the science and roadmap to advance the field. J. Clin. Oncol..

[6] Welch HG, Black WC (1997). Using autopsy series to estimate the disease ‘reservoir’ for ductal carcinoma in situ of the breast: how much more breast cancer can we find?. Ann. Intern. Med..

[7] Page DL, Dupont WD, Rogers LW, Jensen RA, Schuyler PA (1995). Continued local recurrence of carcinoma 15-25 years after a diagnosis of low grade ductal carcinoma in situ of the breast treated only by biopsy. Cancer.

[8] Page DL, Dupont WD, Rogers LW, Landenberger M (1982). Intraductal carcinoma of the breast: follow-up after biopsy only. Cancer.

[9] Collins LC (2005). Outcome of patients with ductal carcinoma in situ untreated after diagnostic biopsy: results from the Nurses’ Health Study. Cancer.

[10] Erbas B, Provenzano E, Armes J, Gertig D (2006). The natural history of ductal carcinoma in situ of the breast: a review. Breast Cancer Res. Treat..

[11] Sagara Y (2015). Survival benefit of breast surgery for low-grade ductal carcinoma in situ: a population-based cohort study. JAMA Surg..

[12] Pilewskie M (2016). Do LORIS trial eligibility criteria identify a ductal carcinoma in situ patient population at low risk of upgrade to invasive carcinoma?. Ann. Surg. Oncol..

[13] Elshof LE (2015). Feasibility of a prospective, randomised, open-label, international multicentre, phase III, non-inferiority trial to assess the safety of active surveillance for low risk ductal carcinoma in situ—The LORD study. Eur. J. Cancer.

[14] Youngwirth LM, Boughey JC, Hwang ES (2017). Surgery versus monitoring and endocrine therapy for low-risk DCIS: the COMET trial. Bull. Am. Coll. Surg..

[15] Lakhani, S.R., Ellis. I.O., Schnitt, S.J., Tan, P.H. & van de Vijver, M. J. *WHO Classification of Tumours of the Breast.* 4th edn (IARC Press, 2012).

[16] Elston CW, Ellis IO (1991). Pathological prognostic factors in breast cancer. I. The value of histological grade in breast cancer: experience from a large study with long-term follow-up. Histopathology.

[17] Cutler SJ, Black MM, Friedell GH, Vidone RA, Goldenberg IS (1966). Prognostic factors in cancer of the female breast. II. Reproducibility of histopathologic classification. Cancer.

[18] Le Doussal V (1989). Prognostic value of histologic grade nuclear components of Scarff-Bloom-Richardson (SBR). An improved score modification based on a multivariate analysis of 1262 invasive ductal breast carcinomas. Cancer.

[19] Silverstein MJ (2003). The University of Southern California/Van Nuys prognostic index for ductal carcinoma in situ of the breast. Am. J. Surg..

[20] Pinder SE (2010). A new pathological system for grading DCIS with improved prediction of local recurrence: results from the UKCCCR/ANZ DCIS trial. Br. J. Cancer.

[21] Rodriques SG (2019). Slide-seq: a scalable technology for measuring genome-wide expression at high spatial resolution. Science.

[22] Ståhl PL (2016). Visualization and analysis of gene expression in tissue sections by spatial transcriptomics. Science.

[23] Roth A (2014). PyClone: statistical inference of clonal population structure in cancer. Nat. Methods.

[24] Takaku M (2018). GATA3 zinc finger 2 mutations reprogram the breast cancer transcriptional network. Nat. Commun..

[25] Samuels Y (2004). High frequency of mutations of the PIK3CA gene in human cancers. Science.

[26] Zardavas D (2018). Tumor PIK3CA genotype and prognosis in early-stage breast cancer: a pooled analysis of individual patient data. J. Clin. Oncol..

[27] Sun J, Nishiyama T, Shimizu K, Kadota K (2013). TCC: an R package for comparing tag count data with robust normalization strategies. BMC Bioinforma..

[28] Zhou Y (2019). Metascape provides a biologist-oriented resource for the analysis of systems-level datasets. Nat. Commun..

[29] Bombonati A, Sgroi DC (2011). The molecular pathology of breast cancer progression. J. Pathol..

[30] Abdel-Fatah TMA (2007). High frequency of coexistence of columnar cell lesions, lobular neoplasia, and low grade ductal carcinoma in situ with invasive tubular carcinoma and invasive lobular carcinoma. Am. J. Surg. Pathol..

[31] Tsuda H (2009). Gene and chromosomal alterations in sporadic breast cancer: correlation with histopathological features and implications for genesis and progression. Breast Cancer.

[32] Buerger H (1999). Comparative genomic hybridization of ductal carcinoma in situ of the breast—evidence of multiple genetic pathways. J. Pathol..

[33] Miligy IM (2019). The clinical and biological significance of HER2 over-expression in breast ductal carcinoma in situ: a large study from a single institution. Br. J. Cancer.

[34] Allred DC (1992). Overexpression of HER-2/neu and its relationship with other prognostic factors change during the progression of in situ to invasive breast cancer. Hum. Pathol..

[35] Latta EK, Tjan S, Parkes RK, O’Malley FP (2002). The role of HER2/neu overexpression/amplification in the progression of ductal carcinoma in situ to invasive carcinoma of the breast. Mod. Pathol..

[36] Jang MH (2012). FGFR1 is amplified during the progression of in situto invasive breast carcinoma. Breast Cancer Res..

[37] Park K, Han S, Kim HJ, Kim J, Shin E (2006). HER2 status in pure ductal carcinoma in situ and in the intraductal and invasive components of invasive ductal carcinoma determined by fluorescence in situ hybridization and immunohistochemistry. Histopathology.

[38] Lari SA, Kuerer HM (2011). Biological markers in DCIS and risk of breast recurrence: a systematic review. J. Cancer.

[39] Pang J-MB (2017). Breast ductal carcinoma in situ carry mutational driver events representative of invasive breast cancer. Mod. Pathol..

[40] Pellacani D, Tan S, Lefort S, Eaves CJ (2019). Transcriptional regulation of normal human mammary cell heterogeneity and its perturbation in breast cancer. EMBO J..

[41] Chou J, Provot S, Werb Z (2010). GATA3 in development and cancer differentiation: cells GATA have it!. J. Cell. Physiol..

[42] Pei X-H (2009). CDK inhibitor p18(INK4c) is a downstream target of GATA3 and restrains mammary luminal progenitor cell proliferation and tumorigenesis. Cancer Cell.

[43] Kouros-Mehr H (2008). GATA-3 links tumor differentiation and dissemination in a luminal breast cancer model. Cancer Cell.

[44] Dydensborg AB (2009). GATA3 inhibits breast cancer growth and pulmonary breast cancer metastasis. Oncogene.

[45] Shahi P (2017). GATA3 targets semaphorin 3B in mammary epithelial cells to suppress breast cancer progression and metastasis. Oncogene.

[46] Mehra R (2005). Identification of GATA3 as a breast cancer prognostic marker by global gene expression meta-analysis. Cancer Res..

[47] Cancer Genome Atlas Network. (2012). Comprehensive molecular portraits of human breast tumours. Nature.

[48] Hruschka N (2020). The GATA3 X308_Splice breast cancer mutation is a hormone context-dependent oncogenic driver. Oncogene.

[49] Koren S (2015). PIK3CAH1047R induces multipotency and multi-lineage mammary tumours. Nature.

[50] Van Keymeulen A (2015). Reactivation of multipotency by oncogenic PIK3CA induces breast tumour heterogeneity. Nature.

[51] Lin CY (2019). Genomic landscape of ductal carcinoma in situ and association with progression. Breast Cancer Res. Treat..

[52] Li H, Durbin R (2009). Fast and accurate short read alignment with Burrows-Wheeler transform. Bioinformatics.

[53] McKenna A (2010). The genome analysis toolkit: a MapReduce framework for analyzing next-generation DNA sequencing data. Genome Res..

[54] Cibulskis K (2013). Sensitive detection of somatic point mutations in impure and heterogeneous cancer samples. Nat. Biotechnol..

[55] Yoshida K (2011). Frequent pathway mutations of splicing machinery in myelodysplasia. Nature.

[56] Wang K, Li M, Hakonarson H (2010). ANNOVAR: functional annotation of genetic variants from high-throughput sequencing data. Nucleic Acids Res..

[57] Favero F (2015). Sequenza: allele-specific copy number and mutation profiles from tumor sequencing data. Ann. Oncol..

[58] Liberzon A (2015). The molecular signatures database (MSigDB) hallmark gene set collection. Cell Syst..

[59] Langfelder P, Horvath S (2008). WGCNA: an R package for weighted correlation network analysis. BMC Bioinforma..

[60] Gierisch JM (2014). Prioritization of research addressing management strategies for ductal carcinoma in situ. Ann. Intern. Med..

