# Supplementary material for: Genomic profiling reveals heterogeneous populations of ductal carcinoma in situ of the breast
Source: Commun Biol. 2021 Apr 1;4:438. doi: 10.1038/s42003-021-01959-9 (PMC8016951; doi:10.1038/s42003-021-01959-9)
Supplement: Supplementary file 1 — Supplementary Information [file 42003_2021_1959_MOESM1_ESM.pdf]

Supplementary Information

Genomic profiling reveals heterogeneous populations of ductal carcinoma in situ of the breast

Satoi Nagasawa[1,2,3]†, Yuta Kuze[1]†, Ichiro Maeda[4,5], Yasuyuki Kojima[2], Ai Motoyoshi[2], Tatsuya Onishi[3], Tsuguo Iwatani[3], Takamichi Yokoe[3], Junki Koike[6], Motohiro Chosokabe[6], Manabu Kubota[6], Hibiki Seino[6], Ayako Suzuki[1], Masahide Seki[1], Katsuya Tsuchihara[7], Eisuke Inoue[8], Koichiro Tsugawa[2], Tomohiko Ohta[9], Yutaka Suzuki[1]*

[1]Department of Computational Biology and Medical Sciences, Graduate School of Frontier Sciences, The University of Tokyo, 5-1-5, Kashiwanoha, Kashiwa-shi, Chiba 277-8561, Japan

[2]Division of Breast and Endocrine Surgery, Department of Surgery, St. Marianna University School of Medicine, 2-16-1, Sugao, Miyamae-ku, Kawasaki 216-8511, Japan

[3]Department of Breast Surgery, National Cancer Center Hospital East, 6-5-1, Kashiwanoha, Kashiwa, Chiba 277-8577, Japan

[4]Department of Diagnostic Pathology, Kitasato University Kitasato Institute Hospital, 5-9-1, Shirokane, Minato-ku, Tokyo 108-8642, Japan

[45]Department of Pathology, Kitasato University School of Medicine, 1-15-1, Kitasato, Minami-ku, Sagamihara 252-0374, Japan

[6]Department of Pathology, St. Marianna University School of Medicine, 2-16-1, Sugao, Miyamae-ku, Kawasaki 216-8511, Japan

[7]Division of Translational Informatics, Exploratory Oncology Research and Clinical Trial Center, National Cancer Center, Kashiwa 277-8577 Chiba, Japan

[8]Showa University Research Administration Center, Showa University, 1-5-8, Hatanodai, Shinagawa-ku, Tokyo 142-8555, Japan

[9]Department of Translational Oncology, St. Marianna University Graduate School of Medicine, 2-16-1, Sugao, Miyamae-ku, Kawasaki 216-8511, Japan

†These authors contributed equally

*Correspondence: Dr. Yutaka Suzuki, Department of Computational Biology and Medical Sciences, Graduate School of Frontier Sciences, The University of Tokyo, 5-1-5, Kashiwanoha, Kashiwa-shi, Chiba 277 8561, Japan. E-mail: ysuzuki@edu.k.u-tokyo.ac.jp, Phone No.: +81-4-7136-3607, Fax No: +81-4-7136-3607

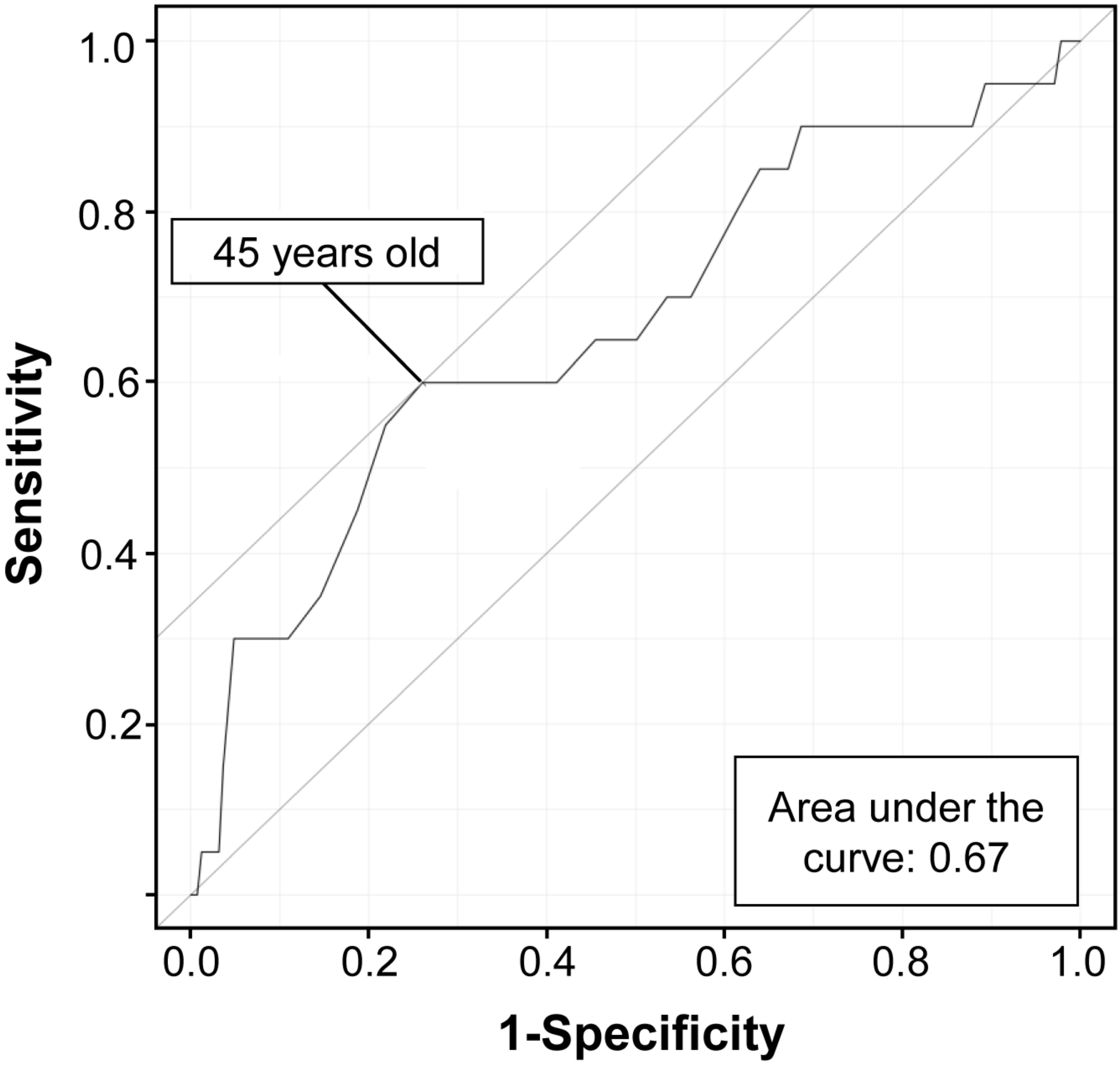

Supplementary Fig. S1 | The receiver operating characteristic (ROC) curve for age
The resulting ROC curve, with an area under the curve (AUC) of 0.67, reveals a poor prognosis. According to the AUC, we selected 45 years as the cutoff (<45 years vs. ≥45 years).

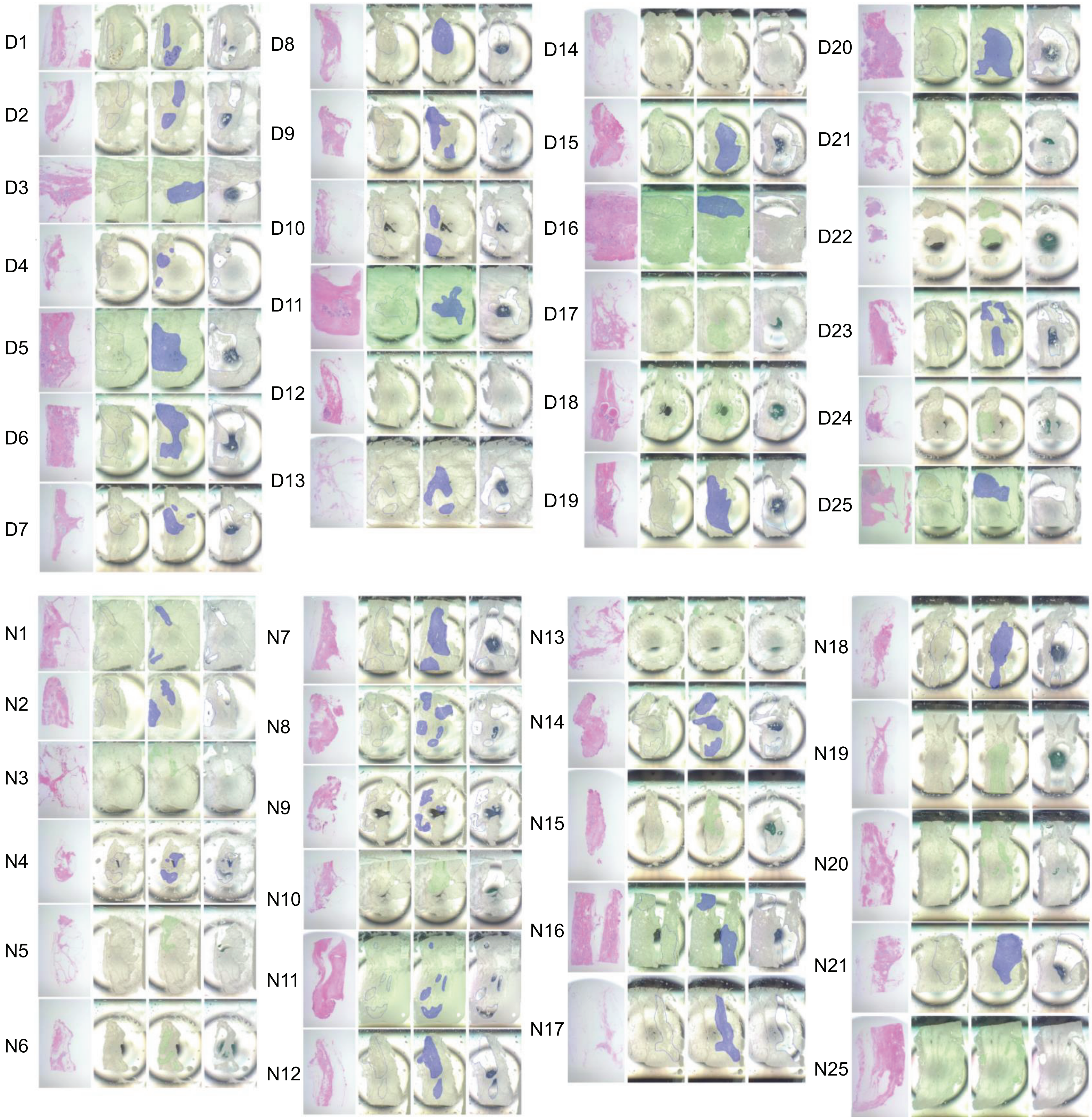

Supplementary Fig. S2 | Microdissection of 25 pure ductal carcinoma in situ (DCIS) specimens in the discovery cohort

Specimens were subjected to careful microdissection to avoid contamination with cells. The first panel from the left shows hematoxylin and eosin staining. The second panel shows the specimens before microdissection. The third panel shows the target region of microdissection. The rightmost panel shows the remaining specimen after microdissection. D, DCIS; N, normal

Supplementary Figure S3

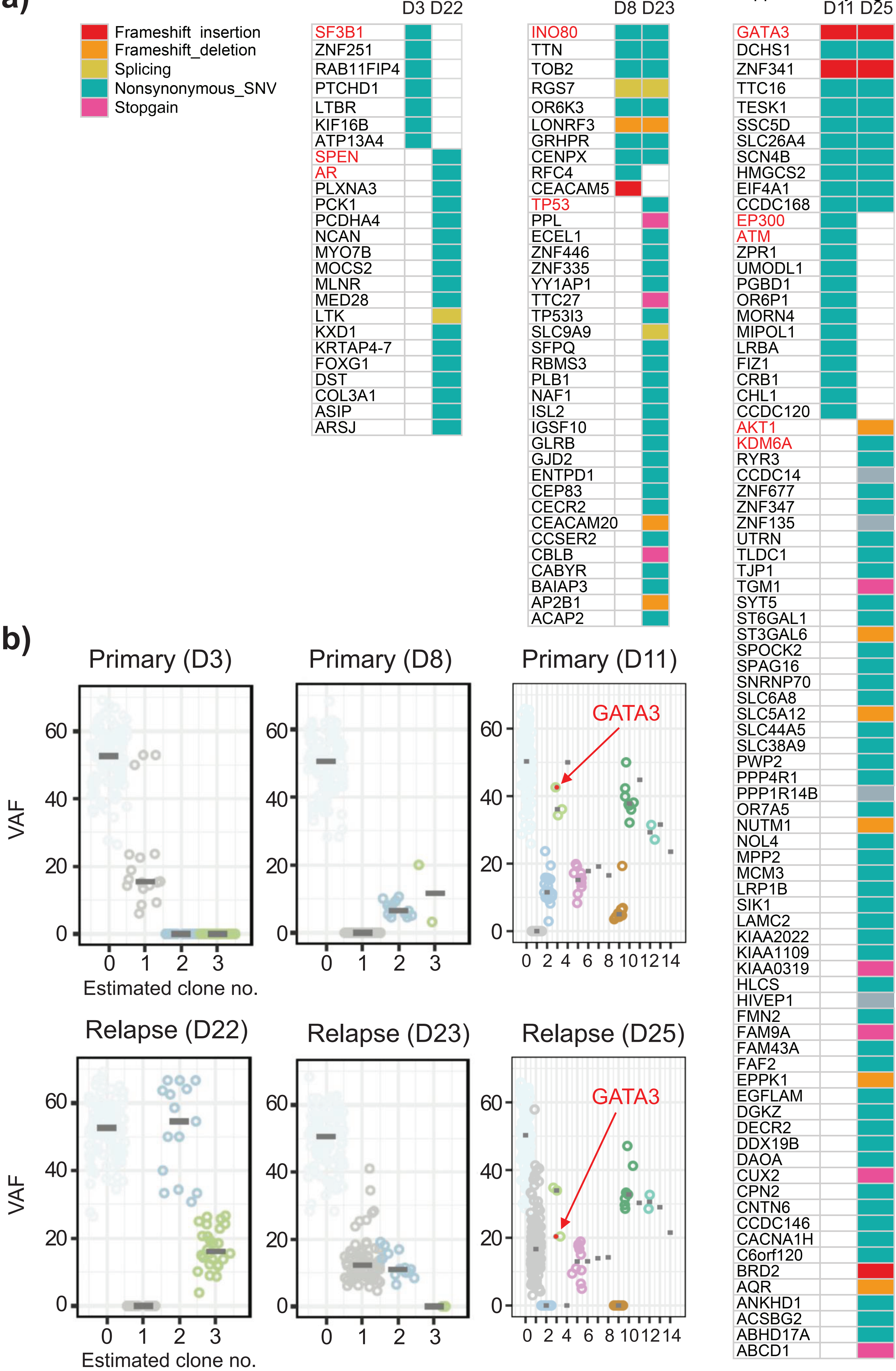

Supplementary Fig. S3 |Comparison of mutations between primary and relapse lesions

S3A: Comparisons of mutations via whole-exome sequencing of primary (D3, D8, and D11) and matched relapse lesions (D22, D23, and D25) are shown. Well-known genes that cause breast cancer are colored red. No foundation mutation was shared by Cases D3 (primary lesion) and D22 (relapse lesion). Therefore, we assumed that recurrence in D3 was indeed a de novo breast cancer (new primary) rather than a recurrence of the primary ductal carcinoma in situ lesion. Contrarily, cases D8 and D11 (matched relapse lesions were labeled D23 and D25, respectively) shared foundation mutations. This suggested that a subclone in the primary lesion also existed in the relapse lesion. Therefore, we assumed that the recurrences in D8 and D11 were indeed relapses of breast cancer (true recurrence).

S3B: The results of subclone analysis performed using PyClone software are shown.

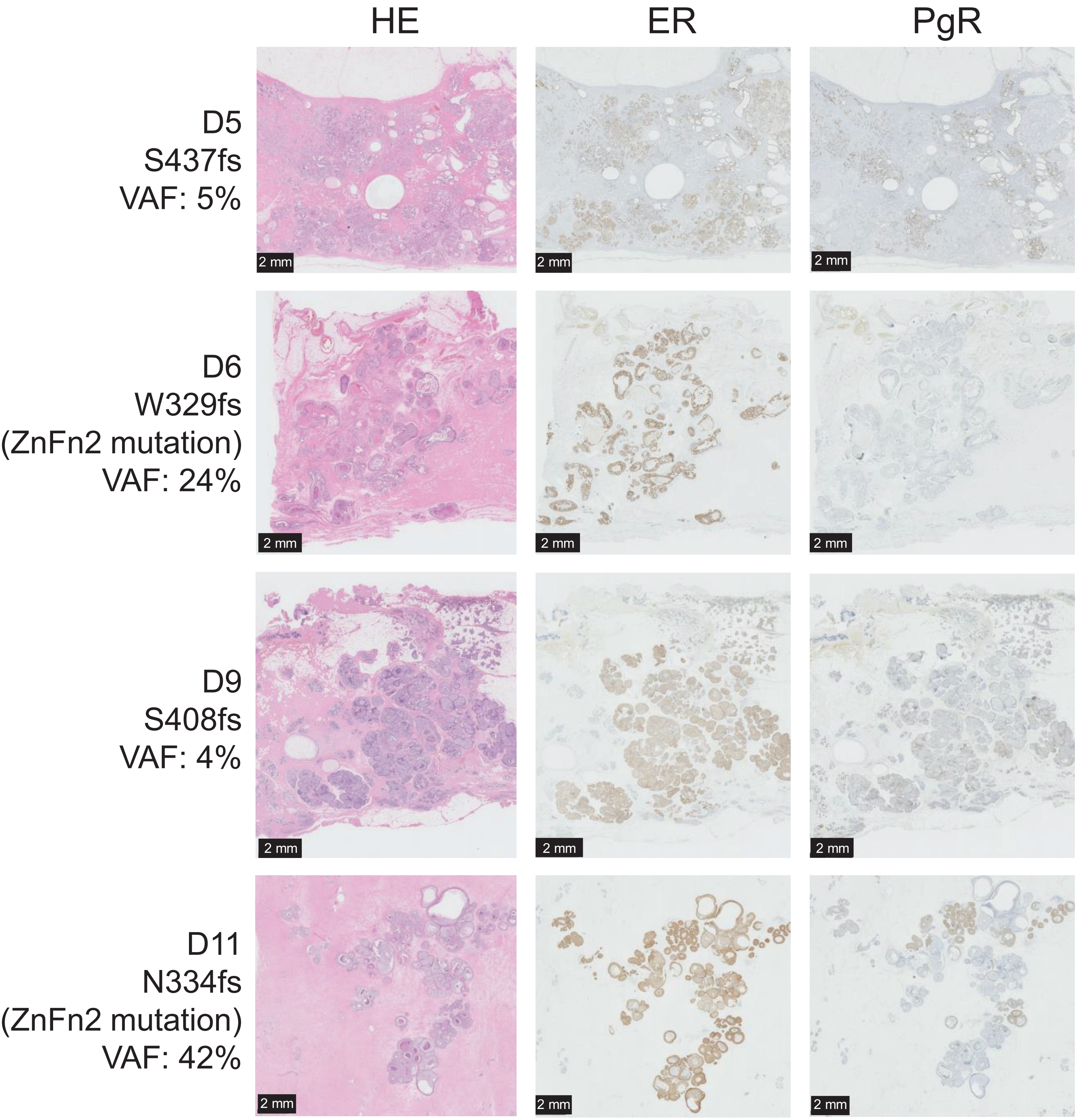

Supplementary Fig. S4 | Progesterone receptor (PgR) expression in GATA3 mutation-positive ductal carcinoma in situ

The panel presents hematoxylin and eosin, estrogen receptor (ER), and PgR staining for patients D5, D6, D9, and D11 with GATA3 mutation. Although ER expression was positive, PgR expression was downregulated in all cases. Reflecting the variant allele frequency of GATA3 mutation, some heterogeneity in PgR expression was observed. Scale bars: 2 mm.

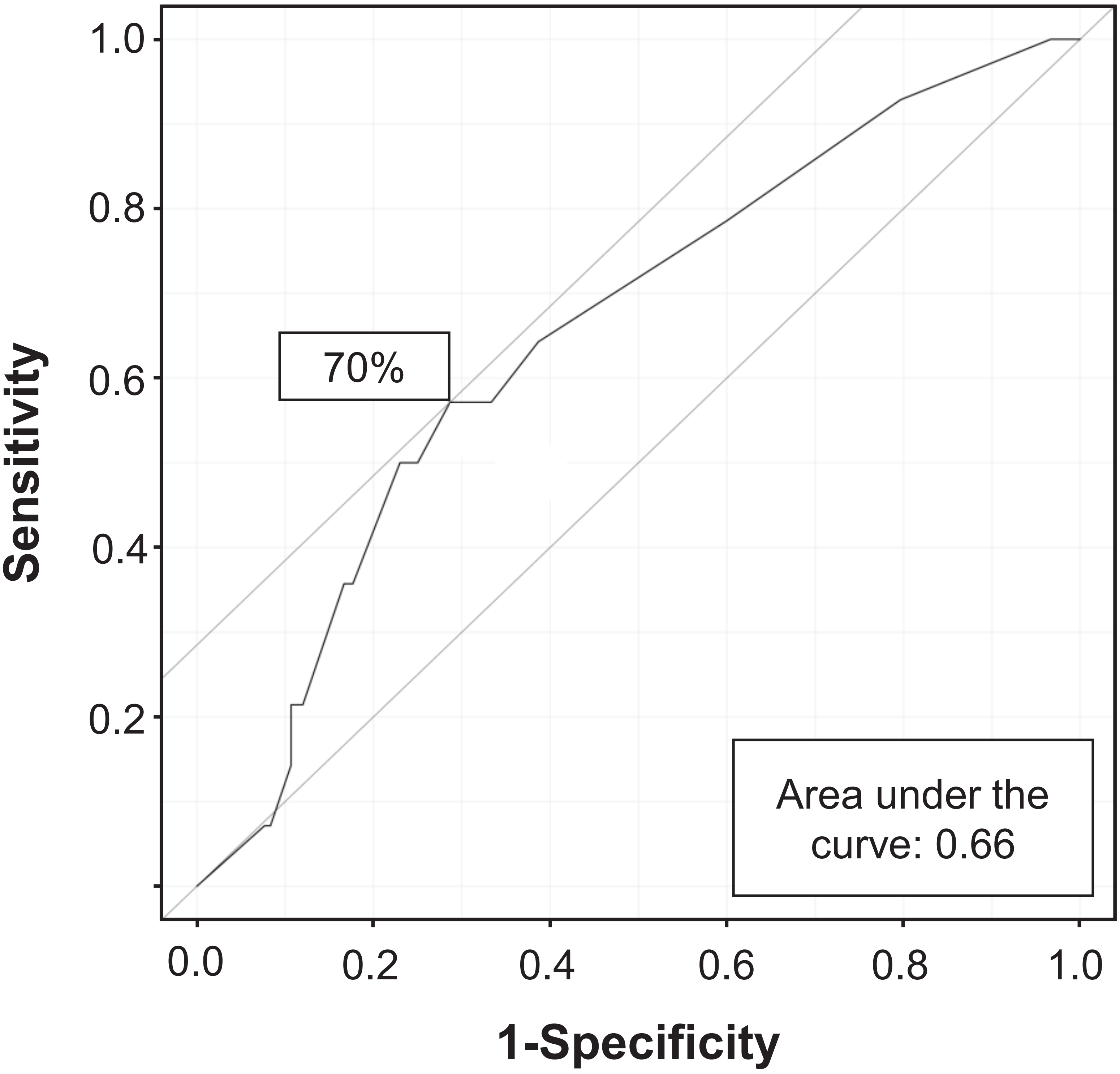

Supplementary Fig. S5 | Receiver operating characteristic (ROC) curve for progesterone receptor (PgR) expression
The resulting ROC curve, which had an area under the curve of 0.66, denoted poor prognosis. Therefore, we decided that PgR expression in 70% or more of cells represented high expression, whereas expression in 60% or fewer cells indicated low expression.

Supplementary Figure S6

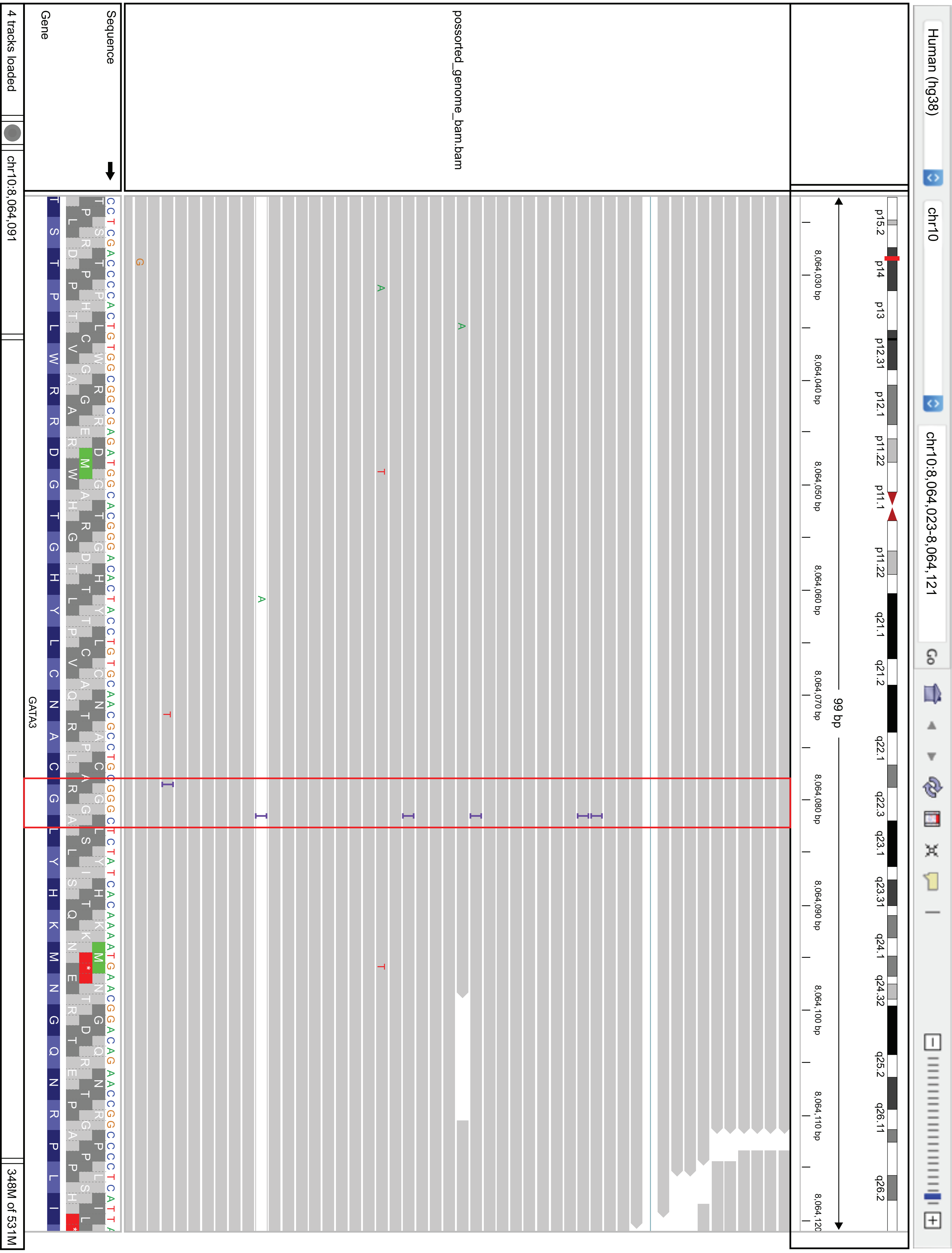

Supplementary Fig. S6 |Selection of reads with GATA3 mutation using the Integrative Genomics Viewer

Spatial transcriptome sequencing (STseq) reads on GATA3 are shown. In Case A, the STseq reads revealed a GATA3 mutation (exon4:c.865dupG:p.C288fs, indicated by a red square), and the mutation was located relatively near the 3′-end of mRNA.

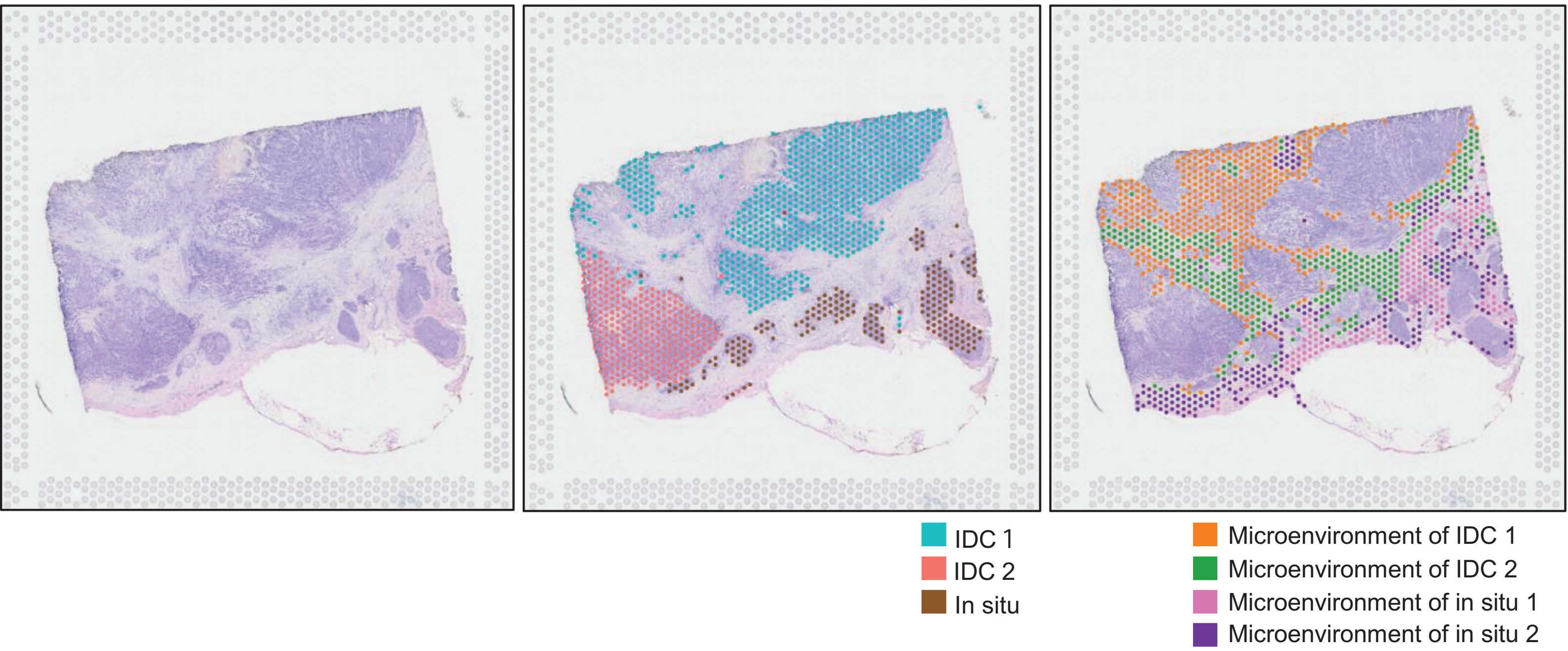

Supplementary Fig. S7 | Visualization of the Visium results of Case B
Hematoxylin and eosin staining (left). Cancer spots were classified into three clusters via un-hierarchical k-means clustering (k = 9, middle). Non-cancer spots (microenvironment) were classified into four clusters (right).

Supplementary Figure S8

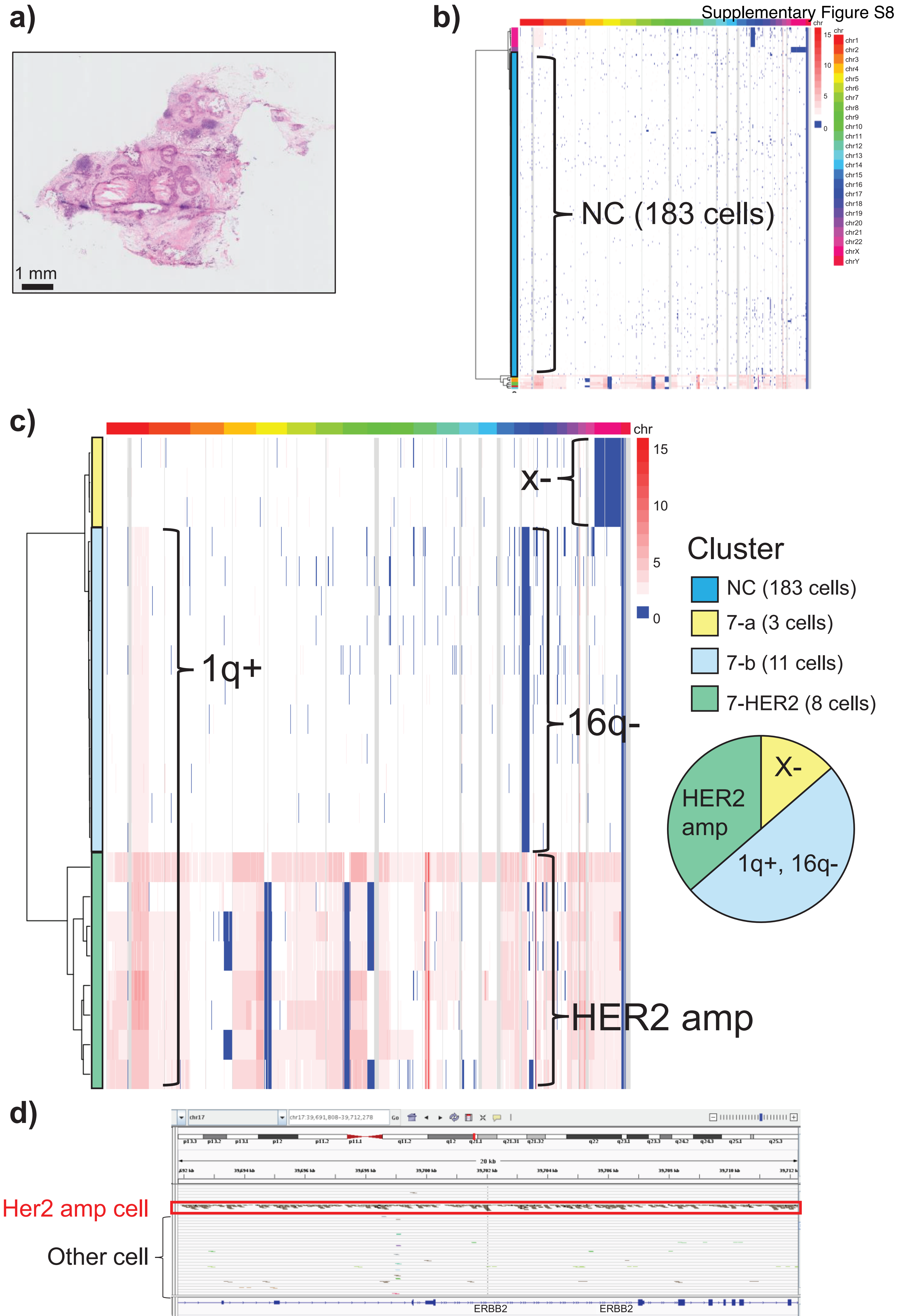

Supplementary Fig. S8| Single-cell copy number variation analysis of a malignant case (Case 7)
a) Hematoxylin and eosin staining for Case 7. Case 7 was a 48-year-old woman (estrogen receptor, 0%; progesterone receptor, 0%; human epidermal growth factor receptor 2 immunohistochemistry score, 3+; Ki-67 index, 50%) who harbored a microinvasion. Therefore, this case was considered a typical case of malignancy. No mutation was detected in PIK3CA or GATA3. The scale bar is 1 mm.
b) Heatmap presents the copy number variation at the single-cell level. The color scale for copy number changes is shown on the right margin. The corresponding chromosomal locations are also shown. A total of 205 cells are represented. The clustering was performed as described in the Materials and Methods. The generated clusters were designated as indicated. The non-cancer cell cluster was designated NC. The remaining cancer cells were further divided into subclusters as shown in (c).
c) Magnification of the cancer populations in (b). The cancer cells were further divided into three major subclusters. The observed three major subclusters were designated clusters 7-a, 7-b, and 7-HER2. Subcluster 7-a (three cells) featured chrX−. Subcluster 7-b (11 cells) harbored 1q+ and 16q−. Subcluster 7-HER2 was further divided into subclones. Note the only subcluster 7-HER2 displayed genomic human epidermal growth factor receptor 2 (HER2) amplification.
The pie chart on the right margin represents the composition of the cellular populations harboring the indicated genomic mutations.
d) Single-cell DNA sequencing reads of the HER2 region are shown using the Integrative Genomics Viewer. Red squares represent cells with HER2 amplification.

Supplementary Figure S9

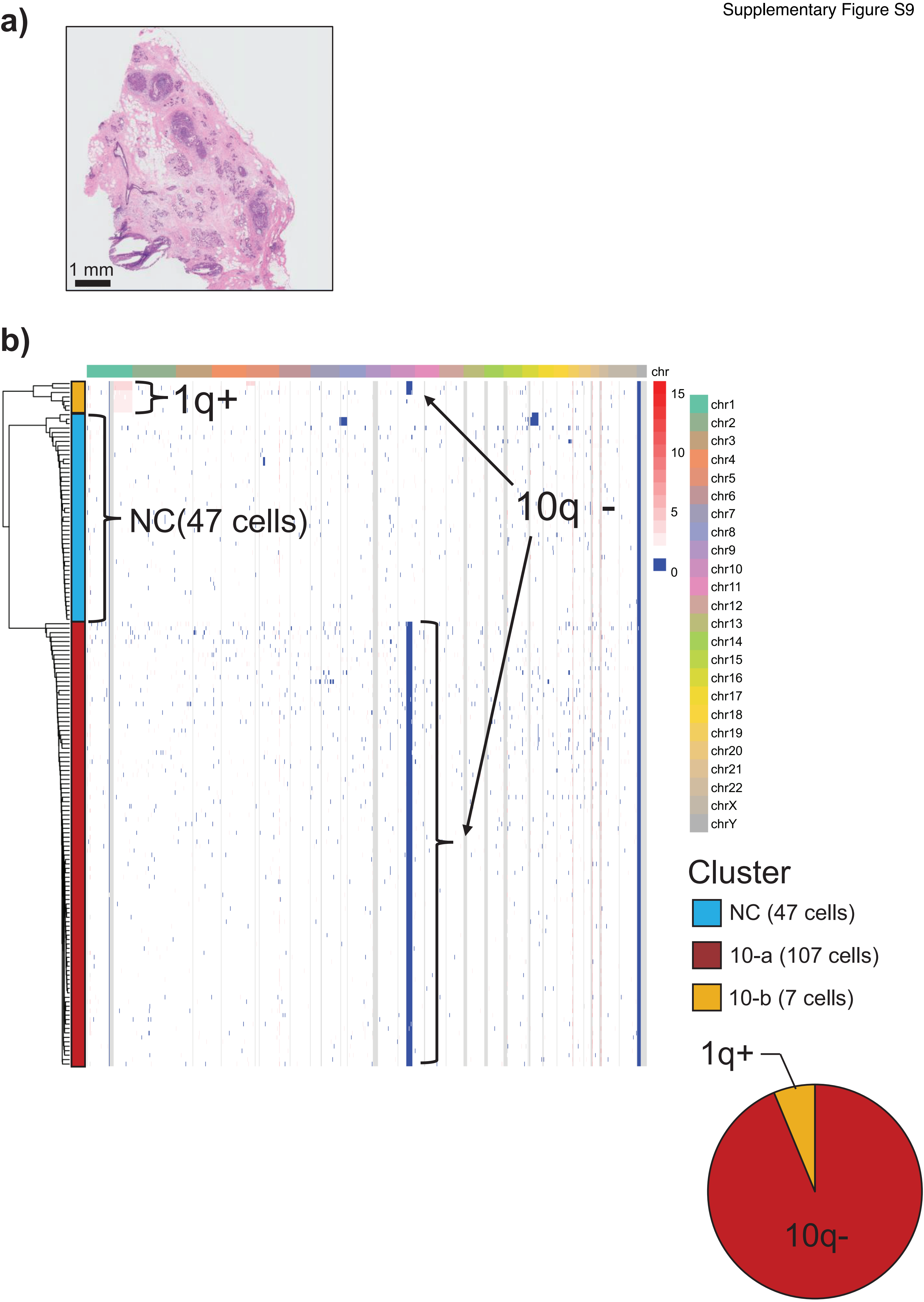

Supplementary Fig. S9| Single-cell copy number variation analysis in a patient without relapse (Case 10)

a) Hematoxylin and eosin staining in Case 10. Case 10 was a 47-year-old woman (ER, 99%; PgR, 99%; human epidermal growth factor receptor 2 immunohistochemistry score, 2+ (FISH 1.1); Ki-67 index, 1-9%) who did not harbor a microinvasion. No mutation was detected in PIK3CA and GATA3. The scale bar is 1 mm.

b) Heatmap presents the copy number variation at the single-cell level. A total of 161 cells are represented. The non-cancer cell cluster was denoted by NC. The cancer cells were further divided into two subclusters, including one major (subcluster 10-a) and one minor subcluster (subcluster 10-b). Subcluster 10-a (107 cells) harbored 10q−. Subcluster 10-b (seven cells) harbored 1q+. Because some clones in subcluster 10-b featured same structural variants as subcluster 10-a, all of the cancer cells were considered to comprise a single cluster.

The pie chart on the right margin represents the composition of the cellular populations having the indicated genomic mutations.

a

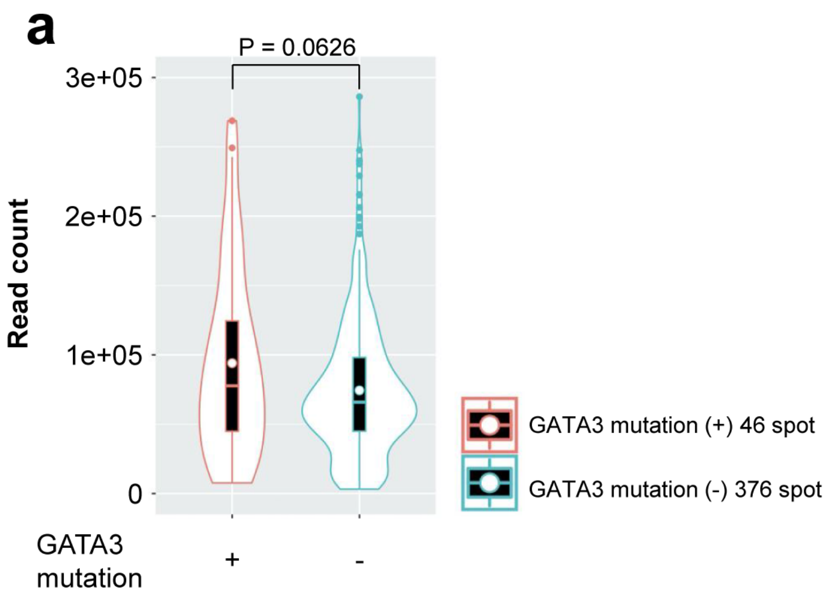

b

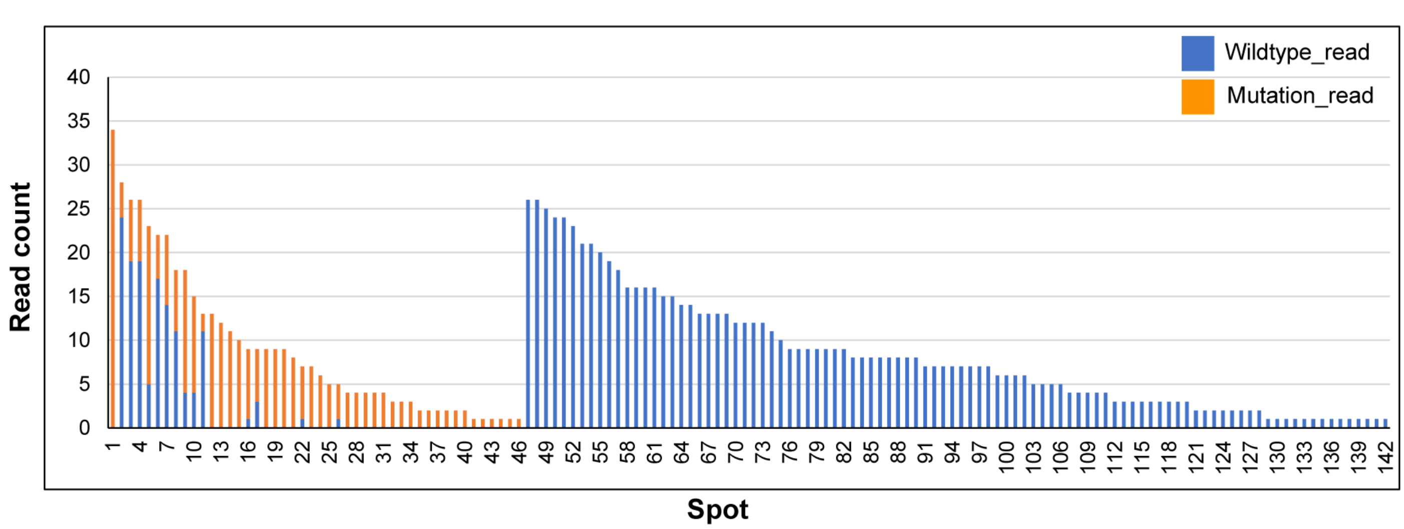

Supplementary Figurel S10
a) Comparison of the total number of UMI reads between 46 spots with and 376 spots without GATA3 mutant reads.
b) Distribution of the number of reads at each mRNA base position (NM_1002295.2) in 422 spots with GATA3 reads. The vertical red line represents the GATA3 mutation site (exon4:c.865dupG).
c) Distribution of the number of GATA3 reads in 142 spots with reads at the GATA3 mutation site (position of the red line in a). GATA3 mutant reads are orange (mutation_read), and reads without GATA3 mutations (wildtype_read) are blue.

Supplementary Table S1 | Clinicopathologic characteristics of patients in the discovery and validation cohorts

Characteristic	Cohort 2 (Discovery cohort) (N = 21)	Cohort 3 (Validation cohort) (N = 72)	Difference (P)
Age at diagnosis			
Median age (range), years	42 (29–76)	49 (34–76)	0.3292
≥45 years, n (%)	9 (42.9)	47 (65.3)	
<45 years n (%)	12 (57.1)	25 (34.7)	
Management, n (%)			
BCS	10 (47.6)	49 (68.1)	0.2099
Mastectomy	4 (19.0)	10 (13.9)	
NSM	7 (33.3)	13 (18.0)	
ER status, n (%)			
Negative	5 (23.8)	20 (27.8)	0.7875
Positive	16 (76.2)	52 (72.2)	
PgR status, n (%)			
Negative	6 (28.6)	23 (31.9)	>0.9999
Positive	15 (71.4)	49 (68.1)	
HER2 amplification, n (%)			
No	13 (61.9)	40 (55.6)	0.8027
Yes	8 (38.1)	32 (44.4)	
Nuclear atypia, n (%)			
Low grade	6 (28.6)	14 (19.4)	0.0956
Intermediate grade	7 (33.3)	43 (59.7)	
High grade	8 (38.1)	15 (20.8)	
Comedo necrosis, n (%)			
No	8 (38.1)	30 (41.7)	0.8066
Yes	13(61.9)	42 (58.3)	
Median tumor size(range), cm	3.0 (1.2–13.4)	2.1 (0.1–10.5)	0.04928

BCS, breast-conserving surgery; NSM, nipple-sparing mastectomy; ER, estrogen receptor; PgR, progesterone receptor; HER2, human epidermal growth factor receptor 2

Supplementary Table S2 | PgR expression by immunohistochemistry with and without GATA3 mutation in 16 ER-positive case

	PgR low	PgR high	
GATA3 mt(+)	4	0	4
GATA3 mt(-)	3	9	12
	7	9	16

mt, mutation; PgR, progesterone receptor

Supplementary Table S3 | Gene list of target sequence.

ABCD1	CDK12	FBXO22	KRAS	NTRK3	RYR3
ABL1	CDK4	FGFR1	LOC389895	OK7K1P	SCN4B
ACIN1	CDK6	FGFR2	LONRF3	OR6K3	SETBP1
ACTN4	CDKN1B	FGFR3	LTK	PALB2	SETD2
AKT1	CDKN2A	FGFR4	MAP2K1	PBRM1	SF3B1
AKT2	CELSR2	FLT3	MAP2K2	PCSK5	SLC26A4
AKT3	CHD7	FOXA1	MAP2K4	PDGFRA	SLC2A12
ALK	CHEK2	GATA3	MAP3K1	PDGFRB	SMAD1
APC	CLCA2	GNA11	MAP3K4	PIK3CA	SMAD4
AR	COL11A2	GNAQ	MDM2	PIK3R1	SMARCA4
ARAF	COL6A1	GNAS	MDM4	PIK3R2	SMARCB1
ARID1A	CREBBP	GRHL3	MED12	POLD1	SMO
ARID2	CRKL	GRHPR	MERTK	PPL	SOX9
ARR3	CTCF	HIRIP3	MET	PPP2R1A	SPEN
ATM	CTNNB1	HMGCS2	MLH1	PRKCI	SPTB
AXIN1	CUL3	HRAS	MSH2	PTCH1	SSC5D
AXL	DCHS1	IDH1	MTOR	PTEN	STAT3
BAP1	DDR2	IDH2	MYC	RAC1	STK11
BARD1	DNMT3A	IGF1R	MYCN	RAC2	STRA13
BCL2L11	ECEL1	IGF2	MYH13	RAD51C	TBX3

BRAF	EGFR	INO80	NF1	RAD54L	TESK1
BRCA1	EIF4A1	JAK1	NFE2L2	RAF1(CRAF)	TOB2
BRCA2	ENO1	JAK2	NOTCH1	RB1	TP53
C2CD4A	EP300	JAK3	NOTCH2	RBMXL3	TRRAP
CBFB	EPHB4	KDM5C	NOTCH3	RET	TSC1
CCDC14	ERBB2	KDM6A	NRAS	RGS7	TTC16
CCDC168	ERBB3	KDR	NRG1	RHOA	TTN
CCND1	ERBB4	KEAP1	NT5C2	ROS1	TYRO3
CD274(PDL1)	ESR1	KIT	NTRK1	RUNX1	VHL
CDH1	EZH2	KMT2A	NTRK2	RUVBL1	ZNF341

Supplementary Table S4 | Clinical information of Visium cases

	Case A	Case B	Case C
Age at diagnosis	41	56	43
Management	Mastectomy	Mastectomy	Mastectomy
Follow up(year)	5.5	4.8	3.2
ER status	Positive (70%)	Positive (90%)	Positive (90%)
PgR status	Positive (90%)	Negative (0%)	Positive (90%)
HER2 amplification (FISH)	3+ (n.a.)	2+ (1.14)	2+ (1.1)
Nuclear atypia in situ)	Intermediate	Intermediate	Low
Comedo necrosis	positive	positive	negative
Tumor size (cm)	1.2	6.5	2.9
GATA3 mutation (VAF)	exon4:c.865dupG:p.C288fs(7%)	-	-
PIK3CA mutation (VAF)	exon5:c.T1035A:p.N345K (26.1%)	exon21:c.A3140T:p.H1047L (40%)	exon10:c.G1633A:p.E545K (25%) exon10:c.A1634G:p.E545G (25%)

ER, estrogen receptor, PgR, progesterone receptor; HER2, human epidermal growth factor receptor 2; FISH, fluorescence in situ hybridization; VAF, variant allele frequency; n.a., not available
