# Supplementary material for: Genomic profiling reveals heterogeneous populations of ductal carcinoma in situ of the breast
Source: Commun Biol. 2021 Apr 1;4:438. doi: 10.1038/s42003-021-01959-9 (PMC8016951; doi:10.1038/s42003-021-01959-9)
Supplement: Supplementary file 2 — Description of Additional Supplementary Files [file 42003_2021_1959_MOESM2_ESM.pdf]

Description of Additional Supplementary Files

File name: Supplementary Data 1

Description: Source data for Table 1.

File name: Supplementary Data 2

Description: Summary of sequence stats.

File name: Supplementary Data 3

Description: Mutation list of Visium Cases.

File name: Supplementary Data 4

Description: Sequence stats of Visium cases.

File name: Supplementary Data 5

Description: Pathway Enrichment analysis of Visume cases.

File name: Supplementary Data 6

Description: Weighted gene co-expression network analysis (WGCNA) and DEG list of CaseC.

File name: Supplementary Data 7

Description: The full list of non-synonymous variants in discovery cohort.

File name: Supplementary Data 8

Description: The full list of non-synonymous variants in validation cohort.
