# Supplementary material for: Genomic profiling reveals heterogeneous populations of ductal carcinoma in situ of the breast
Source: Commun Biol. 2021 Apr 1;4:438. doi: 10.1038/s42003-021-01959-9 (PMC8016951; doi:10.1038/s42003-021-01959-9)
Supplement: Supplementary file 11 — Reporting Summary [file 42003_2021_1959_MOESM11_ESM.pdf]

nature research

Corresponding author(s): Yutaka Suzuki

Last updated by author(s): Feb 18, 2021

# Reporting Summary

Nature Research wishes to improve the reproducibility of the work that we publish. This form provides structure for consistency and transparency in reporting. For further information on Nature Research policies, see our Editorial Policies and the Editorial Policy Checklist.

## Statistics

For all statistical analyses, confirm that the following items are present in the figure legend, table legend, main text, or Methods section.

| n/a | Confirmed | |
|---|---|---|
| ☐ | ☒ | The exact sample size (*n*) for each experimental group/condition, given as a discrete number and unit of measurement |
| ☐ | ☒ | A statement on whether measurements were taken from distinct samples or whether the same sample was measured repeatedly |
| ☐ | ☒ | The statistical test(s) used AND whether they are one- or two-sided<br>*Only common tests should be described solely by name; describe more complex techniques in the Methods section.* |
| ☐ | ☒ | A description of all covariates tested |
| ☐ | ☒ | A description of any assumptions or corrections, such as tests of normality and adjustment for multiple comparisons |
| ☐ | ☒ | A full description of the statistical parameters including central tendency (e.g. means) or other basic estimates (e.g. regression coefficient) AND variation (e.g. standard deviation) or associated estimates of uncertainty (e.g. confidence intervals) |
| ☐ | ☒ | For null hypothesis testing, the test statistic (e.g. *F*, *t*, *r*) with confidence intervals, effect sizes, degrees of freedom and *P* value noted<br>*Give P values as exact values whenever suitable.* |
| ☒ | ☐ | For Bayesian analysis, information on the choice of priors and Markov chain Monte Carlo settings |
| ☒ | ☐ | For hierarchical and complex designs, identification of the appropriate level for tests and full reporting of outcomes |
| ☒ | ☐ | Estimates of effect sizes (e.g. Cohen's *d*, Pearson's *r*), indicating how they were calculated |

*Our web collection on statistics for biologists contains articles on many of the points above.*

## Software and code

Policy information about availability of computer code

| | |
|---|---|
| Data collection | The Visium platform of 10x Genomics (Pleasanton, CA, USA) was used in Spatial transcriptome sequence(STseq), which is a recently developed barcoding-based spatial transcriptomics technology. In STseq, raw FASTQ files and histology images were processed using Space Ranger software v1.0.0 (https://support.10xgenomics.com/spatial-gene-expression/software/pipelines/latest/installation). To visualize spatial expression using histological images, the raw Visium files for each sample were read into Loupe Browser software v4.0.0 (https://support.10xgenomics.com/spatial-gene-expression/software/downloads/latest). |
| Data analysis | Clinical statistics: GraphPad Prism v8.0 and R version 3.5.0.<br>Whole exome and target sequencing analysis: Burrows-Wheeler Aligner Mem (ver 0.7.17), Picard Tools V2.18.25, MuTect2, Genomon pipeline, PyClone program version 0.13, Sequenza.<br>ST seq: Space Ranger software v1.0.0, Loupe Browser software v4.0.0 |

For manuscripts utilizing custom algorithms or software that are central to the research but not yet described in published literature, software must be made available to editors and reviewers. We strongly encourage code deposition in a community repository (e.g. GitHub). See the Nature Research guidelines for submitting code & software for further information.

## Data

Policy information about availability of data

All manuscripts must include a data availability statement. This statement should provide the following information, where applicable:

- Accession codes, unique identifiers, or web links for publicly available datasets
- A list of figures that have associated raw data
- A description of any restrictions on data availability

All sequencing data and pathological images for STseq have been deposited in the DNA Data Bank of Japan under the accession number JGAS00000000202.

# Field-specific reporting

Please select the one below that is the best fit for your research. If you are not sure, read the appropriate sections before making your selection.

☒ Life sciences ☐ Behavioural & social sciences ☐ Ecological, evolutionary & environmental sciences

For a reference copy of the document with all sections, see nature.com/documents/nr-reporting-summary-flat.pdf

# Life sciences study design

All studies must disclose on these points even when the disclosure is negative.

| | |
|---|---|
| Sample size | No statistical methods were used to determine sample size since this is an exploratory study.<br>The sample size for clinical data reported in the table1 is n = 431 patients, which was determined based on the number of patients available retrospectively during the enrollment period between 1/Jan/2007 and 31/Dec/2012. All main conclusions derived from sequencing data were based on sufficient sequencing depth and subject to robustness analyses. |
| Data exclusions | No data were excluded intentionally. Samples with insufficient amount of DNA for sequencing were excluded from this study. |
| Replication | The results of WES were further tested using independent cohorts. |
| Randomization | As this study is observational, no randomization was employed. |
| Blinding | As this study is observational, blinding was not employed. |

# Reporting for specific materials, systems and methods

We require information from authors about some types of materials, experimental systems and methods used in many studies. Here, indicate whether each material, system or method listed is relevant to your study. If you are not sure if a list item applies to your research, read the appropriate section before selecting a response.

## Materials & experimental systems

| n/a | Involved in the study | |
|---|---|---|
| ☒ | ☐ | Antibodies |
| ☒ | ☐ | Eukaryotic cell lines |
| ☒ | ☐ | Palaeontology and archaeology |
| ☒ | ☐ | Animals and other organisms |
| ☐ | ☒ | Human research participants |
| ☒ | ☐ | Clinical data |
| ☒ | ☐ | Dual use research of concern |

## Methods

| n/a | Involved in the study | |
|---|---|---|
| ☒ | ☐ | ChIP-seq |
| ☒ | ☐ | Flow cytometry |
| ☒ | ☐ | MRI-based neuroimaging |

## Human research participants

Policy information about studies involving human research participants

| | |
|---|---|
| Population characteristics | DCIS patients from 3 cohorts were included.<br>The exact characteristics of each cohort are summarized in Table1 and Supplementary tableS1.<br>Raw data for table1 is available from SourceData1. |
| Recruitment | This study was done retrospectively and no patients were directly recruited. |
| Ethics oversight | This study was approved (approval number: 2297-i103) by the Clinical Ethics Committee of St. Marianna University, and a waiver of consent was granted for the use of archival clinical samples from the Department of Pathology. |

Note that full information on the approval of the study protocol must also be provided in the manuscript.
